# Oncolytic adenovirus programmed by synthetic gene circuit for cancer immunotherapy

**DOI:** 10.1038/s41467-019-12794-2

**Published:** 2019-10-22

**Authors:** Huiya Huang, Yiqi Liu, Weixi Liao, Yubing Cao, Qiang Liu, Yakun Guo, Yinying Lu, Zhen Xie

**Affiliations:** 1 MOE Key Laboratory of Bioinformatics and Bioinformatics Division, Center for Synthetic and System Biology, Department of Automation, Beijing National Research Center for Information Science and Technology, Tsinghua University, Beijing, 100084 China; 2 Syngentech Inc., Zhongguancun Life Science Park, Changping District, Beijing, 102206 China; 3 The comprehensive Liver cancer center, The 5th medical center of PLA Genaral Hospital, 100 Xi-Si-Huan Middle Road, Beijing, 100039 China

**Keywords:** Synthetic biology, Cancer immunotherapy, Genetic circuit engineering, Synthetic biology

## Abstract

Improving efficacy of oncolytic virotherapy remains challenging due to difficulty increasing specificity and immune responses against cancer and limited understanding of its population dynamics. Here, we construct programmable and modular synthetic gene circuits to control adenoviral replication and release of immune effectors selectively in hepatocellular carcinoma cells in response to multiple promoter and microRNA inputs. By performing mouse model experiments and computational simulations, we find that replicable adenovirus has a superior tumor-killing efficacy than non-replicable adenovirus. We observe a synergistic effect on promoting local lymphocyte cytotoxicity and systematic vaccination in immunocompetent mouse models by combining tumor lysis and secretion of immunomodulators. Furthermore, our computational simulations show that oncolytic virus which encodes immunomodulators can exert a more robust therapeutic efficacy than combinatorial treatment with oncolytic virus and immune effector. Our results provide an effective strategy to engineer oncolytic adenovirus, which may lead to innovative immunotherapies for a variety of cancers.

## Abstract (web summary)

It is difficult to improve the efficacy of oncolytic virotherapy due to immune system responses and limited understanding of population dynamics. Here the authors use synthetic biology gene circuits to control adenoviral replication and release of immunomodulators in hepatocellular carcinoma cells.

## Introduction

Recent clinical success has demonstrated the great potential of cancer immunotherapy to overcome tumor-mediated immunosuppression. However, the development of immunotherapeutic antibodies and chimeric antigen receptor (CAR)-T cells are limited by the lack of targetable tumor antigens^1,2^. In addition, therapeutic efficacy of individual immunomodulators such as cytokines, chemokines and checkpoint inhibitors is often compromised when infiltrated lymphocytes are limited in tumor microenvironment^3^. Systemic treatment of immunomodulators either alone or in combinations frequently cause severe side effect^4^. These studies highlight a continuous advancement and a large unmet demand in cancer immunotherapy to explore efficient approaches to locally modulate immunological state in tumor microenvironment with reduced side effect.

Oncolytic virus is an attenuated or engineered virus that can cause cancer cell lysis due to selective replication, and trigger systematic immune responses, which has been considered as a class of medicines for cancer immunotherapy^5^. Although many oncolytic viruses alone only cause weak immune responses, therapeutic efficacy of oncolytic virus against tumors can be greatly enhanced by encoding and locally releasing cytokines and chemokines, which helps overcoming immunosuppression in tumor microenvironment with reduced side effect compared to systematic administration of immunomodulators^6–9^. In addition, recent study shows that oncolytic virus (Talimogene laherparepvec) that encodes human granulocyte-macrophage colony-stimulating factor (GM-CSF) can dramatically increase the objective response rate of programmed death protein 1 (PD-1) inhibitor therapy (pembrolizumab) independent on the baseline CD8^+^ lymphocyte infiltration, highlighting the potential of oncolytic virus to change and reactivate the local immune microenvironment against tumors^10^. However, it is still a great challenge to increase tumor-targeting specificity and immuno-stimulation efficacy of oncolytic virus.

The cancer-targeting specificity of oncolytic virus is also critical for the safety and efficacy of oncolytic virus immunotherapy. A variety of viruses have been engineered as oncolytic viruses, such as adenovirus, herpes simplex virus (HSV), and vaccinia virus^6,11,12^. Adenovirus that contains a double-stranded linear DNA genome of ~36 kb, becomes attractive due to low pathogenic risk, high genome stability, wide range of tissue tropism and relatively large DNA loading capacity (up to 8.5-kb foreign DNA)^5,7,13^. In the adenoviral genome, the E1 region encodes E1A and E1B proteins, and induction of E1A expression alone may be sufficient to initiate the virus replication in different cancer cells^14^. The selectivity of oncolytic adenovirus (OV) can be improved by simply using cancer-specific promoters or microRNAs to regulate the E1A expression^7,8,15,16^. However, it is still necessary to further reduce the pathogenicity of adenovirus to ensure the safety in clinical applications. An appealing strategy is to combine multiple inputs to further improve the cancer-targeting specificity^17^. In addition, the loading capacity of replication-competent adenoviral vector limits accommodation of sophisticated gene circuits and modifying OV is still costly and inefficient by using a ‘trial-and-error’ method, which demands an effective engineering framework to facilitate the development of OV^18,19^.

**Fig. 1:**
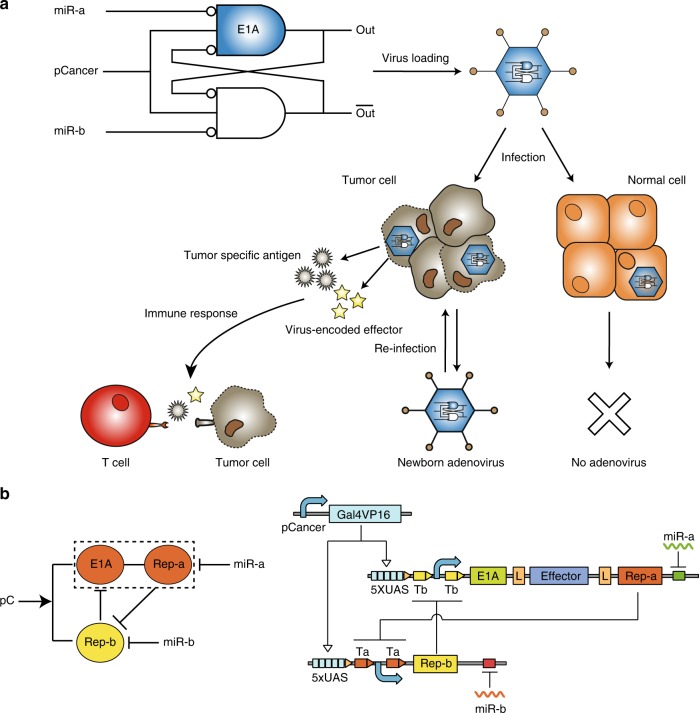
Programmable oncolytic adenovirus by using sensory switch circuit. **a** The schematic diagram of synthetic oncolytic adenovirus. A sensory switch circuit with mutual inhibitions controls the expression of adenoviral E1A gene and immune effector by sensing one promoter and two microRNA inputs, resulting in selective viral replication in cancer cells and modulations of immune responses. pCancer cancer-specific promoter, miR-a a microRNA expresses at a high level in normal cells but at a low level in cancer cells, miR-b a cancer-specific microRNA. The sensory switch circuit produces high levels of E1A and immune effector when the following three conditions are satisfied: pCancer is activated, miR-a is low and miR-b is high. **b** The schematic diagram of sensory switch circuit is shown in the left. A sensory switch circuit consists of a Gal4VP16 activator gene driven by pCancer, and two mutually inhibiting repressor genes controlled by two miRNAs respectively. UAS upstream activation sites, Effector immune effector, L self-cleavage 2A linker, Ta and Tb binding sites of two repressors (Rep-a and Rep-b) respectively

Built on engineering principles, an important aim of mammalian synthetic biology is to construct synthetic gene circuits with modular and standardized parts for sophisticated information processing and programmable regulations inside live cells. Several mammalian gene circuits have been engineered to integrate multiple promoter and microRNA inputs for identification of specific cancer cells and tumor-specific immunomodulatory expression^17,20–25^. Recently, we demonstrate that a gene circuit composed of two mutually inhibiting transcription activator-like effector repressors (TALERs) can function as a robust switch controlled by microRNA inputs^26,27^. To further improve the cancer-targeting specificity of OV and trigger immune response against cancer cells in the cancer microenvironment, we engineer a compact (<8.5-kb) and programmable switch circuit to control the replication of OV and expression of immune effectors (Fig. 1a). In this setup, a transcription activator driven by a cancer-specific promoter turns on two mutually inhibiting repressors that are respectively repressed by two microRNAs (miR-a and miR-b). The switch-controlled E1A and immune effector genes co-express by using a self-cleavage 2A linker when the activator and miRNA-b are at high levels and miRNA-a is at low level (Fig. 1b)^28^.

To realize the application potential of synthetic gene circuits for identification and destroy of cancer cells, it is essential to develop an appropriate delivery vector platform. In this study, we develop a sensory switch circuit which is about 6.5 kb in length and establish a hierarchical assembly method to efficiently load the circuits into adenoviral vector backbone, which can control adenoviral replication and coexpression of immune effectors, such as human GM-CSF, interleukin-2 (IL-2), single-chain variable fragments (scFvs) against either programmed death-1 (PD-1) or programmed death-ligand 1 (PD-L1). It has been shown that viral-encoded immune effectors can promote anti-cancer cytotoxicity of oncolytic virotherapy by modulating tumor microenvironment^29–31^. For proof-of-concept, we aim to construct the programmable sensory switch to target hepatocellular carcinoma (HCC) cells because HCC is the major type of liver cancers with large unmet clinical needs. We demonstrate the modularity of our circuit design by changing immunomodulatory genes, and confirm the high selectivity of programmed oncolytic virus to kill a variety of HCC cancer cells in cell cultures and in mouse models.

Unlike traditional drugs with defined pharmacokinetics and pharmacodynamics, the outcome of oncolytic virus immunotherapy relies on the population dynamics and interactions of tumor cells, viruses and surrounding immune cells, which makes it challenging to systematically understand important features of oncolytic virotherapy. Mathematical models can be helpful to analyze dynamics of complex therapeutic system^32^. Inspired by previous studies^33,34^, we establish coarse-grained models to quantitatively describe the system of oncolytic virotherapy in three different scenarios, where the lymphocytes are absent, or present with or without immunomodulators.

In all, this study assembles programmed OVs with sensory gene circuit switch that exert high selectivity to kill HCC in cell cultures and mouse models. Through mathematical model, this study identifies key characteristics of oncolytic virotherapy and provides guideline for future improvements.

## Results

### Characterization of sensory switch circuits

**Fig. 2:**
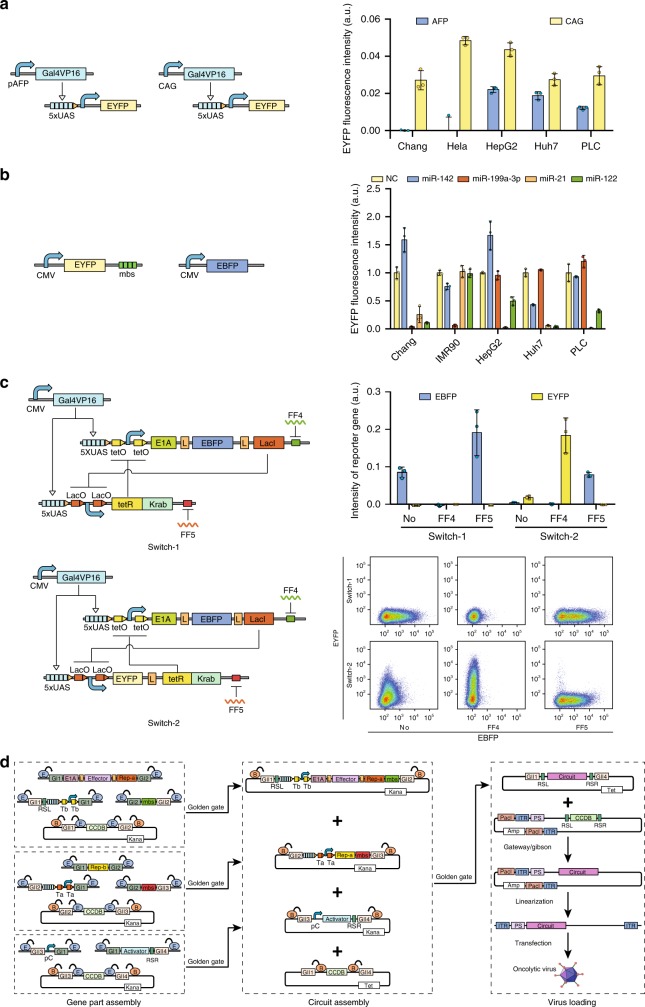
Construction of sensory switch circuit. **a** Functional analysis of AFP promoter (III) in different cell lines by transient transfection. The constitutive CAG promoter was used as a positive control. **b** Functional analysis of microRNAs in indicated cell lines. Four tandem repeats of microRNA binding sites were inserted into the 3′-UTR of EYFP reporter gene. The CMV-driven EBFP was used as an internal control in transient transfection experiments. **c** Characterization of sensory switch circuit in HEK293 cells by transient transfection. LacO binding site of LacI repressor, tetO binding site of tetR: Krab repressor, L self-cleavage 2A linker, FF4 shRNA-FF4, FF5 shRNA-FF5, the right bottom panel shows representative flow cytometry scatter plots measured 48 h after transfection. **d** Hierarchical assembly of sensory switch circuit and loading into adenoviral vector. E in blue circle, Esp3I; B in orange circle, BsaI; L in box, self-cleavage 2A linker; GI1~4 and GII1~4, different overhangs released by Esp3I or BsaI; ccdB, ccdB toxin coding gene; RSL and RSR left and right Gateway recombination site, mbs microRNA binding sites, Kana kanamycin resistance gene, Tet tetracycline resistance gene, Amp ampicillin resistance gene, PacI PacI cutting site, ITR inverted terminal repeat, PS packaging signal. **a**~**c** Each data point shows mean ± s.d. of EYFP or EBFP from three independent replicates. Source data are provided as a Source Data file

Because the loading capacity of replication-competent adenoviral vector is up to ~8.5 kb, we constructed sensory switch circuit by using gene parts with a reduced size. Consistence with previous reports^35^, by using quantitative RT-PCR we confirmed that α-fetoprotein (AFP) gene expressed in several types of HCC cells but not in Chang cells originally derived from normal human liver tissue (Supplementary Fig. 1a). To reduce the length of AFP promoter, we constructed 5 truncated AFP promoter derivatives and analyzed the promoter activity by using transient transfection. We found that the EYFP expression driven by all of these derivatives is high in HepG2 cell which was at least 32-fold higher compared to that in Chang cell (Supplementary Fig. 1b). The derivative III that harbored a G-to-A point mutation and two enhancer elements displayed the strongest promoter activity in a variety of HCC cells but not in Chang cells or HeLa cells, which was used as the liver-cancer-specific promoter in the rest of this study (Fig. 2a). By searching public databases^36^ and performing the microRNA functional assay, we confirmed that miR-21 can be used to distinguish HCC cells from Chang and human Caucasian fetal lung fibroblast (IMR90) cells, while miR-199a-3p was an appropriate microRNA biomarker for normal liver cells which also highly expressed in Chang and IMR90 cells but expressed at low levels in tested HCC cells (Fig. 2b)^36^. To ensure that the sensory circuit cannot be switched on in normal lymphocytes surrounding liver tumors, we further included miR-142 as a biomarker for normal cells because miR-142 has been shown to highly express in the normal haemocytes^37^.

We engineered sensory switches with the activator Gal4VP16 and repressors (LacI and tetR:Krab). The tetR and LacI binding sites were placed downstream of five tandem repeats of upstream activation sites (UASs), flanking a minimal CMV promoter (Fig. 2c). Therefore, LacI that coexpressed along with E1A and enhanced blue fluorescent protein (EBFP) by a self-cleavage 2A linker and tetR:Krab mutually inhibited with each other and were repressed by two synthetic shRNAs (shRNA-FF4 and shRNA-FF5) respectively (Fig. 2c). The *EBFP* gene was served as a fluorescent reporter to evaluate the performance of the sensory switch circuit, which can be flexibly replaced with immunomodulatory genes. We constructed two sensory switch circuits with or without coexpression of the EYFP reporter along with tetR:Krab (Fig. 2c). We demonstrated that both switches can be correctly reset to either state by co-transfecting the corresponding shRNA input into HEK293 cells (Fig. 2c). Based on these results, we chose switch-1 as the founding circuit framework because of the smaller circuit size and a higher E1A induction that may lead to a higher virus replication rate compared to the switch-2.

To facilitate the construction of adenoviral vectors, we established a modular and hierarchical strategy to assemble the switch circuit based on the Golden Gate and Gibson cloning method^38^. In the first round of Golden Gate reaction, different genetic elements including the promoter, coding regions and microRNA binding sites that are selected for targeting specific cancer cells were assembled into three gene parts (Fig. 2d). Similarly, these gene parts were assembled into the switch circuit in the second round of the Golden Gate reaction. Finally, the switch circuit was loaded into the adenoviral vector by using Gateway or Gibson method, which allowed virus packaging after the linearized adenoviral vector was transfected into HEK293 cells (Fig. 2d). We placed the E1A-encoding gene expression unit immediately downstream of the virus packaging signal (PS), followed by the tetR:Krab-encoding and Gal4VP16-encoding gene expression units (Fig. 2d), because we previously demonstrated that switch circuits with a similar architecture function correctly without insulation between gene expression units^39^.

### Functional comparison of sensory switch circuits

**Fig. 3:**
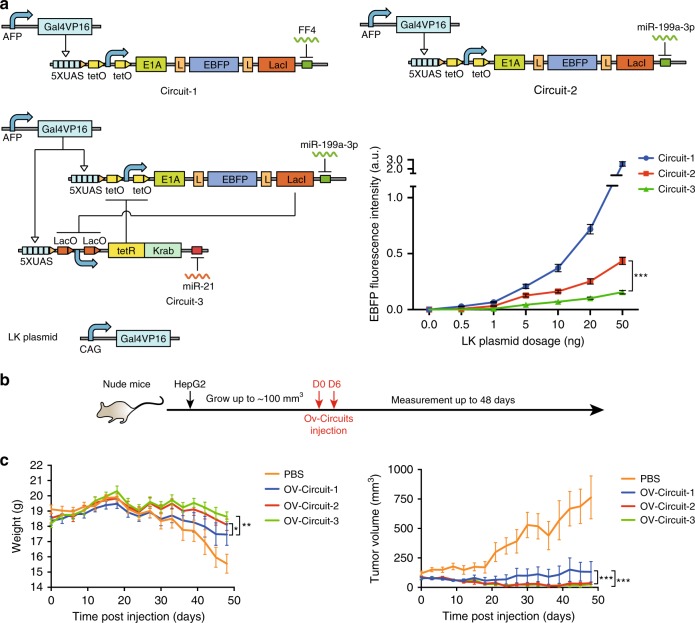
Comparison of the sensory switch circuit with the other switch circuits in vitro and in vivo. **a** Circuits performance in response to leaky expression of Gal4VP16 in vitro. Circuits were co-transfected along with varying amount of the CAG-driven Gal4VP16 (LK plasmid) as leaky expression into HEK293 cells. Each data point shows mean ± s.d. from three independent replicates, *P *<* 0.05, **P *<* 0.01, ***P *<* 0.001. **b** Schematic diagram of in vivo experimental design with xenografted HepG2 nude mouse model. **c** 1 × 10^9^ VP of OV-Circuits were injected into xenografted tumor twice a week right after the size of tumor reached ~100 mm^3^. PBS was used as a negative control. The left panel shows the mouse weight after treatment. Each data point shows the mean ± s.d. weight (n = 10) at indicated day. The right panel shows the xenografted tumor size after treated. Each data point shows the mean ± s.d. tumor size (*n* = 10) at indicated day, *P *<* 0.05, **P *<* 0.01, ***P *<* 0.001,  Student’s *t*-test was performed. Source data are provided as a Source Data file

To assay the specificity and efficacy of the sensory switch circuit (circuit-3) in cell culture and in nude mouse model, we constructed open-loop switch circuits under the control of the promoter only (circuit-1) or both the promoter and microRNA input (circuit-2). To test the response of the sensory switch circuit when the expression of Gal4VP16 was leaky, these three circuits along with varying amount of the CAG-driven Gal4VP16 were transient co-transfected into HEK293 cells respectively (Fig. 3a). In HEK293 cells, the AFP promoter was inactive and the miR-21 level was low, while the miR-199a-3p level was high (Supplementary Fig. 1c). Therefore, adding the CAG-driven Gal4VP16 into HEK293 cells mimicked leaky expression of the AFP promoter. We demonstrated that the circuit-3 was able to tolerate at least 10-fold and 5-fold leaky expression of the AFP promoter than circuit-1 and circuit-2, respectively (Fig. 3a). This result demonstrated that the mutual inhibition circuit had a superior robustness against the promoter leakiness.

Next, we packaged oncolytic adenovirus with these circuits (OV-Circuits) in HEK293 cells, and injected 1 × 10^9^ virus particle (VP) of each OV-circuit into the xenografted HepG2 tumor twice a week right after the size of tumor reached ~100 mm^3^ (Fig. 3b). OV-Circuit-2 and OV-Circuit-3 showed superior efficacy to maintain the mouse weight and reduce the tumor size than OV-Circuit-1, although significant difference was not observed between treatments with OV-Circuit-2 and OV-Circuit-3 (Fig. 3c). Then, we analyzed the DNA and RNA distribution at one week after virus injection by using quantitative PCR or RT-PCR. The DNA of three OV-Circuits enriched only in tumors, at least 171-fold higher than those assayed in other normal cells and tissues (Supplementary Fig. 2a). The RNA level of OV-Circuit-3 was highest among three tested OV-Circuits in tumors, but showed least leaky expression in normal cells and tissues (Supplementary Fig. 2b). Taken together, these results indicated that OV-Circuit-3 was safe and exhibited comparable therapeutic efficacy to OV-Circuit-2.

### Detection of specificity of Synthetic OVs in vitro

**Fig. 4:**
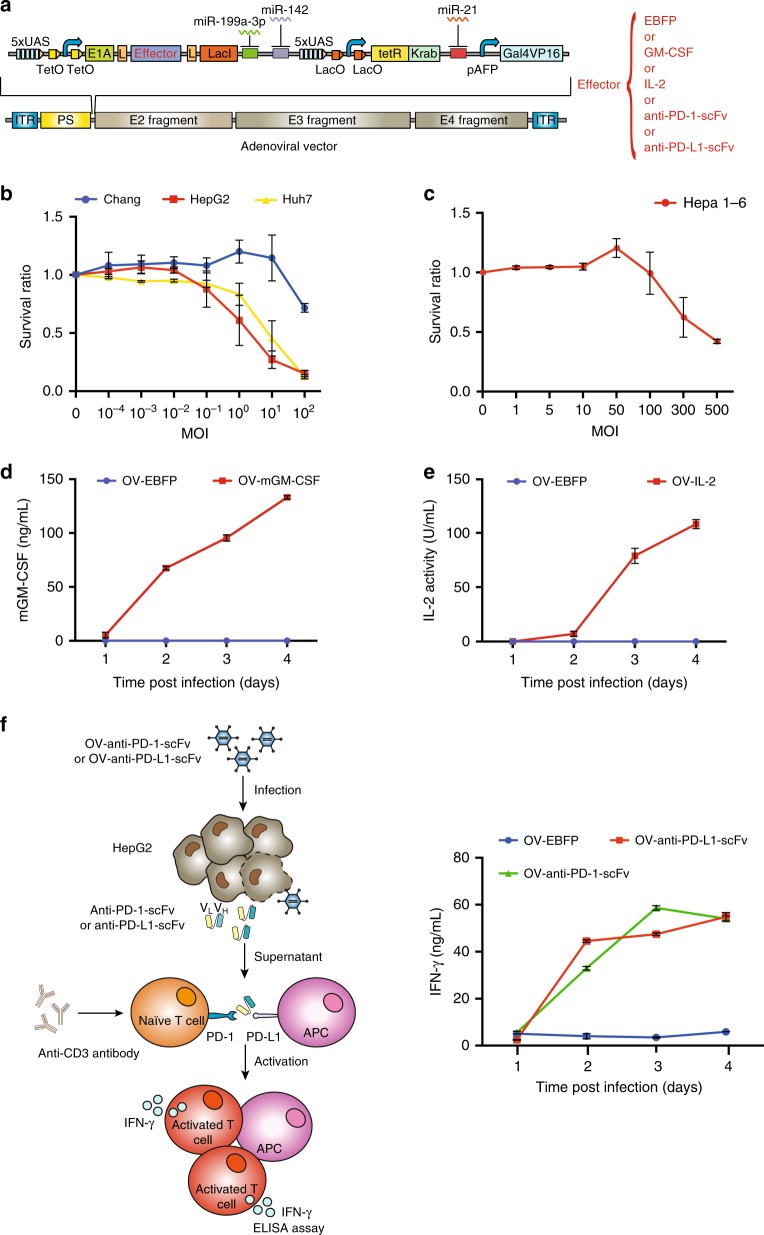
Functional assay of synthetic oncolytic adenovirus in vitro. **a** The schematic diagram of adenoviral genome loaded with sensory switch circuit encoding different immune effectors (OV-Effector). The survival ratios of different human HCC cells in Panel **b** and mouse Hepa1-6 cells in Panel **c** were measured by using MTS cell viability assay 6 days after incubation with indicated amount of OV-EBFP. Data are shown as mean ± s.d. from three independent replicates. **d**–**f** 3 × 10^6^ HepG2 cells were incubated with 10 MOI of oncolytic adenovirus encoding indicated immune effectors for 4 days and the supernatants were collected for measurement of mGM-CSF production by using ELISA in (**d**), or the bioactivity of IL-2 by using MTS proliferation assay on cytokine-dependent cell line CTLL-2 in (**e**), or the bioactivity of anti-PD-1-scFv and anti-PD-L1-scFv by using ELISA in mouse splenocyte system in the presence of anti-CD3 antibody in (**f**). Data are shown as mean ± s.d. from three independent replicates. Source data are provided as a Source Data file

Next, we started to load sensory switch circuits encoding different effectors (OV-Effector) into oncolytic adenoviral vector by using our assembly strategy, and then produced the OV in HEK293 cells. In this circuit setup, the high E1A level can be achieved to trigger adenoviral replication when the following conditions are met in cancer cells: (1) the AFP promoter is turned on; (2) the miR-21 level is high; (3) the miR-199-3p and miR-142 levels are low (Fig. 4a). Firstly, we produced a synthetic oncolytic virus encoding EBFP reporter (OV-EBFP) and assayed the killing efficiency of OV-EBFP on Chang cell and different HCC cells by using cell proliferation assay after incubation with different amounts of viruses for 6 days. We observed that more than 100 multiplex of infection (MOI) of OV-EBFP was required to kill 50% of Chang cells, while only 0.1 or 1 MOI of OV-EBFP was needed to kill half of cultured HepG2 or Huh7 cells (Fig. 4b). Similarly, we found that OV-EBFP displayed ~100-fold more efficient killing effect on two other HCC cells (Hep3B and PLC) than Chang cells (Supplementary Fig. 3). However, ~300 MOI of OV-EBFP were required to efficiently kill the mouse liver-cancer Hepa1-6 cells (Fig. 4c), which may be due to the low AFP gene expression level (Supplementary Fig. 1a) and low viral replication capability in Hepa1-6 cells^40^.

In addition, we replaced the EBFP gene in synthetic oncolytic virus with genes encoding therapeutic proteins to improve local anti-tumor efficacy. Four different virus strains were produced, expressing different immunomodulators, including IL-2, mouse GM-CSF (mGM-CSF), and two scFvs against either PD-1 (anti-PD-1-scFv) or PD-L1 (anti-PD-L1-scFv). To validate the release of immunomodulators in vitro, we infected HepG2 cells with 10 MOI of indicated virus for 4 days and collected the supernatant containing virus-encoded immunomodulators for functional assays. We confirmed that functional GM-CSF and IL-2 can be produced and secreted by using ELISA and an assay with a cytokine-dependent cell line, respectively (Fig. 4d, e). In addition, we demonstrated that the anti-PD-1-scFv and anti-PD-L1-scFv in the corresponding supernatants harvested after adenovirus infections for 2 or 4 days were functional and successfully stimulated high levels of IFN-γ after adding into anti-CD3 stimulated murine splenocytes for 3 days (Fig. 4f).

### Functional assay of synthetic OVs in nude mouse

**Fig. 5:**
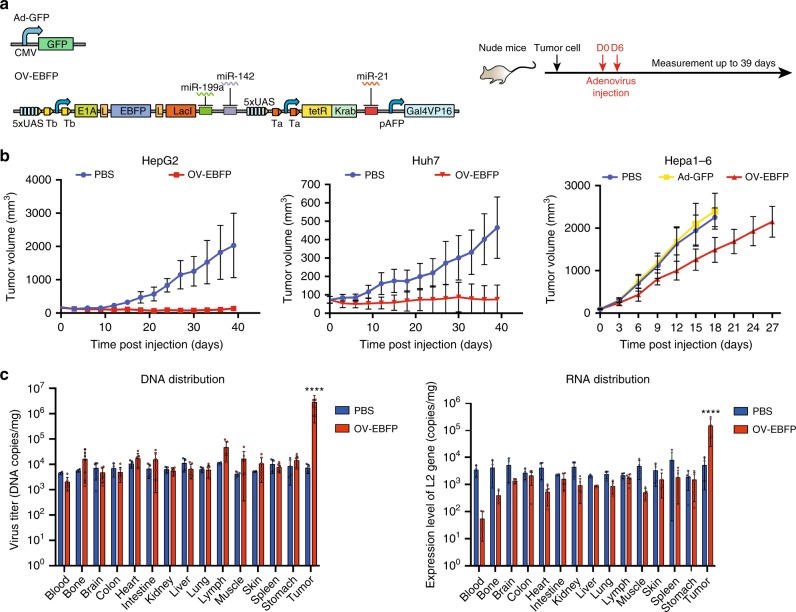
Effect of synthetic oncolytic adenovirus in nude mouse models. **a** The schematic diagram of OV-EBFP and non-replicable adenovirus (Ad-GFP). The experimental design of nude mouse experiments is shown in the right panel. **b** 1 × 10^9^ VP of OV-EBFP or Ad-GFP were injected into indicated xenografted tumor twice a week right after the size of tumor reached ~100 mm^3^. PBS was used as a negative control. Each data point shows the mean ± s.d. tumor size (*n* = 9 or 10) at indicated day. **c** DNA (left) and RNA (right) distribution of OV-EBFP in HepG2 xenografted nude mice 1 week after injection of 1 × 10^9^ VP of OV-EBFP (*n* = 7 in left, *n* = 5 in right) when the size of tumor exceeded ~500 mm^3^. PBS (*n* = 4) was used as a negative control. Data are shown as mean ± s.d. from three independent replicates by using quantitative PCR. Student’s t-test was performed. Data are shown as mean ± s.d., *P *<* 0.05, **P *<* 0.01, ***P *<* 0.001, ****P *<* 0.0001. Source data are provided as a Source Data file

To evaluate the efficacy of synthetic OV in the immune-deficient nude mouse model, we injected 1 × 10^9^ VP of OV-EBFP into the xenografted HepG2 or Huh7 tumor twice a week right after the size of tumor reached ~100 mm^3^ (Fig. 5a). Compared to the mice treated with the mock reagent, the tumor growth in both HepG2 and Huh7 xenograft mice was significantly delayed up to 39 days after the OV-EBFP administrations, indicating that synthetic OV can cause a strong tumor lysis in vivo (Fig. 5b). In a similar experimental setting, we also observed that the Hepa1-6 tumor growth was also impeded after the administration of OV-EBFP but not the non-replicating Ad-GFP virus, although the tumor size of all treated Hepa1-6 model mice exceeded 1600 mm^3^ in 4 weeks (Fig. 5b). These results suggested that the replication capability of oncolytic virus was essential for efficient tumor lysis, and the effect of tumor lysis alone may fail to eliminate tumors when tumor cells grow fast enough to compensate the tumor lysis. We also analyzed the DNA and RNA distribution of OV-EBFP in nude mouse after we injected OV-EBFP into xenografted tumor by using quantitative PCR or RT-PCR. We observed that OV-EBFP DNA mainly enriched in tumors at one week after virus injection, which was at least 55-fold higher in HepG2 tumor and Huh7 tumor than those assayed in other normal tissues (Fig. 5c and Supplementary Fig. 4). At two weeks after virus injection, the OV-EBFP level was still at least fivefold higher in Huh7 tumor than the other normal tissues (Supplementary Fig. 4). Similarly, OV-EBFP RNA were mainly detected in HepG2 tumor (Fig. 5c). These results indicated that the OV-EBFP can target and impede tumors with a high specificity.

### Functional assay of synthetic OVs in immune-competent mice

**Fig. 6:**
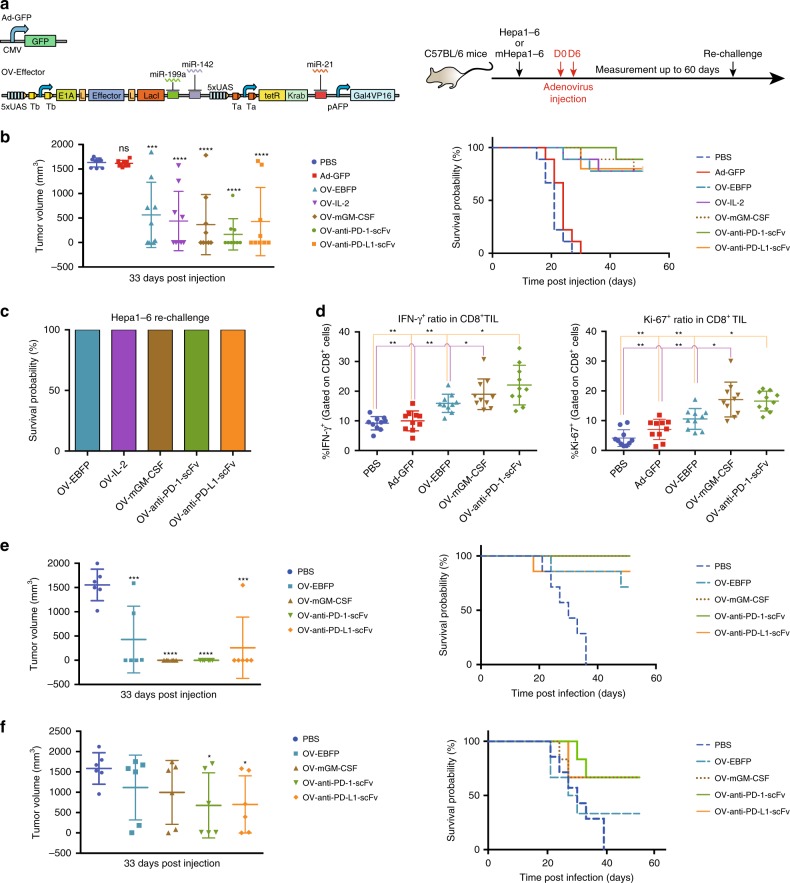
Effect of synthetic oncolytic adenovirus in immune-competent mouse models. **a** The schematic diagram of non-replicable adenovirus (Ad-GFP) and OV-EBFP without the viral backbone. The experimental design of immune-competent mouse experiments is shown on the right. **b** Each mouse was intratumorally injected with 1 × 10^9^ VP of indicated Ad-GFP or OV-Effector twice in one week right after the size of Hepa1-6 tumor reach to 100 mm^3^. PBS was used as a negative control. Tumor volume (left) and survival ratio curve (right) are shown (*n* = 9 or 10) at the 33^rd^ day post injection. **c** Re-challenge survived mouse (*n* = 5 ~ 7) with 1 × 10^6^ Hepa1-6 cells in the contralateral position away from the first transplantation site 60 days after initial treatments. **d** The frequency of IFN-γ^+^ (left panel) and Ki-67^+^ (right panel) cells among tumor infiltrating CD8^+^ T cell in Hepa1-6 bearing mice (*n* = 10) at 14 days after treated with 1 × 10^8^ VP of indicated viruses. **e** 1 × 10^9^ VP of indicated Ad-GFP or OV-Effector were injected into mHepa1-6 bearing mice twice in one week right after the size of tumor reach to 100 mm^3^. PBS was used as a negative control. Tumor volume (left) and survival ratio curve (right) are shown (*n* = 6) at the 33rd day post injection. **f** 1 × 10^7^ VP of indicated Ad-GFP or OV-Effector were injected into mHepa1-6 bearing mice twice in one week right after the size of tumor reach to 100 mm^3^. PBS was used as a negative control. Tumor volume (left) and survival ratio curve (right) are shown (*n* = 6) at the 33rd day post injection. Student’s *t*-test was performed. Data are shown as mean ± s.d., *P *<* 0.05, **P *<* 0.01, ***P *<* 0.001, ****P *<* 0.0001. Source data are provided as a Source Data file

To assay whether co-release of different immunomodulators can promote the efficacy of synthetic oncolytic viruses, we established a subcutaneous Hepa1-6 tumor model by using immune-competent C57BL/6 mice and injected 1 × 10^9^ VP of different synthetic oncolytic viruses into the Hepa1-6 tumor twice a week right after tumor size reached ~100 mm^3^ (Fig. 6a). Similar to our previous observation (Fig. 5b), we found that the non-replicating adenovirus (Ad-GFP) failed to inhibit Hepa1-6 tumor growth, while the replication-competent OV-EBFP caused an obvious delay of tumor growth (Fig. 6b and Supplementary Fig. 5). Compare to the mock and Ad-GFP treatment, all tested OVs induced a robust Hepa1-6 tumor regression leading to durable curing among at least 33.3% of tested mice with OV-EBFP and 66.7% of tested mice with OV-anti-PD-1-scFv treatment at the 33rd day (Fig. 6b left and Supplementary Fig. 5). Especially, over 80% mice survived with no tumor or a durable tumor up to 60 days after treated with synthetic OV expressing anti-PD-1-scFv (Fig. 6b right and Supplementary Fig. 5).

To test whether the survived mice obtain vaccination against the HCC tumor cells after oncolytic virotherapy, we subcutaneously transplanted 1 × 10^6^ Hepa1-6 cells into the contralateral site away from the first transplantation site 60 days after the initial treatment. We observed that all survived mice rejected the second challenge of Hepa1-6 cells, suggesting that immunological memory was induced to prevent tumor metastasis (Fig. 6c). To examine whether these OVs can promote cytotoxic T cell response that is thought to directly cause the clearance of tumor cells, 1 × 10^8^ VP of different synthetic OVs were injected into established Hepa1-6 tumors in C57BL/6 mice. Histopathology studies of tumors revealed that lymphocytes in tumors treated with synthetic OVs were more abundant than tumors treated with the mock reagent or OV-EBFP (Supplementary Fig. 6). By using flow cytometer, we found a greater proportion of IFN-γ^+^ and Ki-67^+^ cells among the tumor filtrating CD8^+^ T cells after the treatment with synthetic OV expressing mGM-CSF or anti-PD-1-scFv compared to the treatment with the OV expressing EBFP or the non-replicating Ad-GFP virus (Fig. 6d). These results indicated that expressing immune stimulators can promote the cytotoxic T cell response during oncolytic virus therapies.

To test whether increasing the transactivation of sensory switch circuits can improve the OV efficacy against tumor, we developed a modified Hepa1-6 cell line (mHepa1-6) that constitutively express Gal4VP16 to activate the coexpression of E1A and immune effector after OV-Effector treatment (Supplementary Fig. 7a). Firstly, the expression level of human GM-CSF (hGM-CSF) in Hepa1-6, HepG2 cell lines and mHepa1-6 mix were analyzed when infected with 10 MOI OV-hGM-CSF for 48 h. We detected a comparable level of hGM-CSF in mixed mHepa1-6 and in HepG2, which is at least 62-fold higher than that in Hepa1-6 at 48 h after virus infection (Supplementary Fig. 7b). Comparable amounts of functional hGM-CSF could be produced and secreted by mHepa1-6 and HepG2 cells after infected with 10 MOI of OV-hGM-CSF for 4 days (Supplementary Fig. 7c). In addition, we found that the killing efficacy of OV-EBFP on mHepa1-6 was much higher than that on Hepa1-6 by using cell proliferation assay after treated with varing amount of viruses for 6 days (Supplementary Fig. 7d).

Next, we tested the efficacy of OV-Effectors against xenografted mHepa1-6 tumor in immune-competent C57BL/6 mouse. When treated with 1 × 10^9^ VP of OV-mGM-CSF and OV-anti-PD-1-scFv for 33 days, the growth of mHepa1-6 tumors in all tested mice were eliminated, leading to a superior survival benefit compared to other treatments (Fig. 6e and Supplementary Fig. 8a). However, the low viral dosage (1 × 10^7^ VP) resulted in a decreased efficacy against mHepa1-6 tumors (Fig. 6f and Supplementary Fig. 8b). These results indicated that the OV with an enhanced transactivation of sensory switch circuits showed an enhanced tumor killing efficiency.

### Modeling of OV therapy

**Fig. 7:**
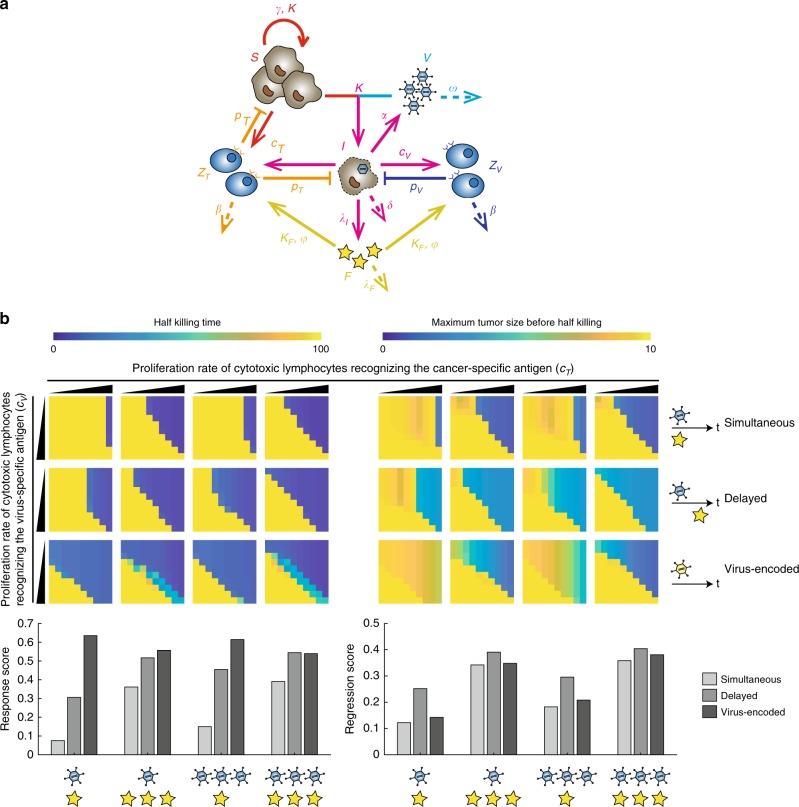
Modeling of oncolytic virus therapeutic system. **a** Schematic diagram of the model with uninfected (*S*) and infected (*I*) cancer cells, free viruses (*V*), cytotoxic lymphocytes recognizing virus-specific antigen (*Z*_*V*_) and cancer-specific antigen (*Z*_*T*_), and immune effectors (*F*). Parameters are explained in “Methods”. **b** Predicted therapeutic efficacy of different administration strategies of replicable virus and immune effector, where immune effector was administrated along with or after the virus injection, or encoded by the virus. The initial amount of virus (MOI = 5 (one virus symbol) or 50 (three virus symbols)) and initial amount or production rate of immune effectors (10 a.u. (one star symbol) or 50 a.u. (three star symbols)) are noted on the bottom. Calculation of half killing time, maximum tumor size before half killing, response score and regression score is explained in “Methods”. Each matrix shows parameter combinations of *c*_*V*_ (10^1.6^ ~ 10^3.5^ a.u.) and *c*_*T*_ (10^1.2^ ~ 10^3.1^ a.u.)

To quantitatively understand the systematic behavior of oncolytic virotherapy that contains complicated feedback regulations and elucidate the influence of the amount of viruses and immune effectors on the therapeutic efficacy, we established coarse-grained models inspired by previous studies, which modeled the dynamics of the amounts of tumor cells, free viruses and cytotoxic lymphocytes in the tumor-immune-virus system^33,34^ (Fig. 7a, Supplementary Figs. 9a and 10a). The minimal model excluded the immune system and only consisted of three components, where free viruses can infect uninfected tumor cells that turned into infected tumor cells (Supplementary Fig. 9a, and “Methods”). Viral degradation was measured (Supplementary Fig. 9b), suggesting that viral clearance was negligible in cell culture and mainly due to infecting normal cells, dilution in body fluid, and degradation by neutralizing antibodies in vivo. We monitored the number of HCC cells incubated with different amount of OV-EBFP over 5 days to fit the minimal model and measure other key parameters (tumor proliferation rate *γ*, viral infection rate *κ*, infected tumor lysis rate *δ* and viral descendant number *α*) in the tumor-virus system (Supplementary Fig. 9c). To evaluate therapeutic efficacy of oncolytic virus with varying parameters, we calculated the half killing time and maximum tumor size before half killing based on our mathematical model (See details in “Methods”). The simulations with the minimal model showed the intuitional result that the efficacy of virus therapy can be dramatically reduced when increasing the virus clearance rate and when the virus lost replication capability (Supplementary Fig. 9d, e). In addition, we focused on the two-sided effect of the immune response on virotherapy and extended the minimal model by including only two types of cytotoxic lymphocytes that recognized either virus-specific antigen or cancer-specific antigen and directly attacking tumor cells. We ignored the regulations from other immune cells and assumed that the lymphocyte responses against cancer-specific antigen were firstly activated by the lysis of infected tumor cells (Supplementary Fig. 10a). We evaluated generally the half killing time and maximum tumor size before half killing among varying strength of both lymphocyte responses as response score and regression score. We found that adding lymphocytes into the system greatly increased the efficacy of oncolytic virotherapy at varying strength of lymphocyte responses against cancer-specific antigen, especially when virus clearance rate was increased and nullify the virotherapy in the minimal model (Supplementary Fig. 10b). Interestingly, unlike the non-replicable virus, a low MOI treatment of oncolytic virus displayed a higher chance (response score) to efficiently kill tumor cells than that with a high MOI treatment in simulations as low MOI might lead to slow clearance of infected cells and increase the activation of lymphocyte response against cancer-specific antigen by the lysis (Supplementary Fig. 10b).

Recent study showed that simultaneous administration of both oncolytic virus and immunomodulator can synergistically enhance therapeutic efficacy^41^. In addition, immunomodulators can also be administrated at a later time point or produced by oncolytic virus. To evaluate the effect of different administration methods on combinatorial immunotherapies, we further extended our model, assuming that immune effectors which were either encoded by oncolytic virus or administrated along with the virus can promote the proliferation of both cytotoxic lymphocytes (Fig. 7a). Similar to our previous observations (Supplementary Figs. 9e and 10b), oncolytic virus displayed a better therapeutic efficacy than non-replicable virus when coupled with immunomodulators by using three different delivery methods (Fig. 7b and Supplementary Fig. 11a). Compared to simultaneous administration, our simulation results showed that administration of immune effector with optimized delay time led to a higher possibility for fast tumor regression (Fig. 7b and Supplementary Fig. 11b). These results suggested that lymphocyte responses towards cancer cells, especially uninfected cancer cells, depend on the lysis of infected cells, and the paradoxical action between oncolytic virus and tumor cells is capable of generating a balanced immune response to efficiently eliminate both tumor cells and virus. Interestingly, the simulated treatment of oncolytic virus that encoded immune effector increased the possibility of fast tumor regression even with the low MOI of oncolytic virus or the low production rate of immune effector, along with the pseudoprogression reported in some cases of immunotherapy^42^, indicating that coupling oncolytic virus with self-encoded immune effector may enhance therapeutic efficacy of oncolytic virus immunotherapy due to feedback control of the immunomodulator production, and effective tumor killing is robust against varying production rates of immune effectors (Fig. 7b and Supplementary Fig. 11c).

## Discussion

In this study, we established a platform to quickly engineer synthetic OV harboring the sensory switch circuit, which allowed the integration of multiple promoter and microRNA inputs to control the viral replication and the release of immune effectors in desired cancer cell type with high selectivity. By using this strategy, the sensory switch circuit can be modified to control expression of multiple immune effectors, which may further increase immune responses against tumor cells. In addition, our sensory switch circuit consists only three components, including one activator Gal4VP16 to initiate the sensory switch and two mutually inhibiting repressors LacI and tetR:Krab (Fig. 1b). The simple circuit design may help reduce efforts for tuning and optimizing gene circuits to obtain desired performance, and the modular design strategy allows flexible modifications and refactoring to target a new type of cancer cells by reusing the core gene parts. Furthermore, our sensory switch circuits can also be adapted to program the targeting specificity of other types of oncolytic viruses such as HSV and poxvirus by tightly regulating expression of key genes required to initiate viral replication^6,43^. However, the promoter activity and microRNA levels may fluctuate in tumor cells among different patients. Even in the same tumor, cell-to-cell variations due to tumor heterogeneity may affect the circuit performance. Although it has been shown that circuit topology with mutual inhibitions can render a robust switch behavior^26,44^, optimization of our sensory switch circuits will be likely necessary to further increase the specificity of oncolytic virus to target desired tumor cells but not normal tissues.

One of successful immunotherapy strategies focuses on inhibiting PD1 and PD-L1 interaction between tumor infiltrated T cells and tumor cells^45^. However, systemic administration of PD1 blockade often results in severe side effects^46^ and even potentially causes T-cell non-Hodgkin’s lymphoma^47^. Another challenging is that a large number of patients display no response to PD1 inhibition therapy when CD8^+^ T cell is absent in tumor microenvironment^3^. Excitingly, systemic anti-PD-1 treatment in combination with intralesional injection of Talimogene laherparepvec is capable of changing tumor microenvironment from an immunologically cold state to an inflamed tumor^10^. Interestingly, our mathematical models predict that changing from simultaneous to sequential administration of oncolytic virus and immune effector can enhance the efficacy of combinatorial therapy, and local injection of oncolytic virus that encodes immune effector by itself can further increase the possibility to yield a high therapeutic efficacy (Fig. 7b). Therefore, local treatment with oncolytic virus that encodes immune effector such as PD-1 blockade is likely an attractive alternative to the combinatorial therapy of oncolytic virus and systemic anti-PD-1 antibody administration, which may also help reducing the side effect caused by PD-1 inhibitors due to controllable and local release of PD-1 blockade. However, our mathematical model is over simplified. More details in the tumor microenvironment were considered in other models^48^, and further experiments are needed to narrow down the parameter ranges and verify these predictions. Nevertheless, our mathematic model is a useful tool to quantitatively understand the systematic behavior of OV therapeutic system, which may help identifying hints to further improve the efficacy of viral immunotherapy alone or in combinations.

Furthermore, we have demonstrated in immune-competent mouse model that combining tumor lysis and secretion of immune effectors during oncolytic virus therapy can have a synergistic effect on promoting local lymphocyte cytotoxicity and systematic vaccination against targeted cancer cells. However, several issues should be addressed before applying this technology in clinical uses. A few non-human proteins (Gal4VP16, LacI and tetR:Krab) are produced in cells after infection with our synthetic OV. Whether these exogenous proteins impair normal cell functions and trigger an immune overreaction remains to be examined. Furthermore, the dynamics of immunomodulators produced by synthetic OV should be monitored, which helps determining a safe dosage window for clinical applications. In summary, programming oncolytic virus by using synthetic gene circuits can increase the specificity of oncolytic virotherapy and enable controllable and local expression of immune effectors, which promises a powerful strategy to treat a variety of cancers.

## Methods

### Cell culture and transfection

HEK293 (293-H) and HEK293FT cell lines were purchased from Life Technologies. Chang cell, human HCC cell lines (HepG2, PLC and Hep3B) and human cervix adenocarcinoma cell line (HeLa) were purchased from ATCC. Mouse HCC cell line (Hepa1-6) was purchased from Procell. The human HCC cell line (Huh7) was purchased from BeNa culture collection Co., Ltd. IMR90 cell was a gift from Xiaowo Wang lab in Tsinghua university. All the cells are routinely maintained. The Chang liver cell line originates from normal liver and has been contaminated by HeLa cells prior to its deposition in ATCC cell bank. In this study we take tha Chang cell as the control cell of HCC cell lines because its high express level of AFP promoter and miR-199a-3p markers.

### Animal models

BALB/c nude mice and C57BL/6 mice were purchased from Beijing Vital River Laboratory Animal Technology Co., Ltd (Beijing, China). The mice were housed under SPF condition in the animal facility at Tsinghua University. These experiments were approved by the Institutional Animal Care and Use Committee of Tsinghua University.

### Reagents and enzymes

Restriction endonuclease, ATP, poly-nucleotide kinase (PNK), T4 DNA ligase, Quick DNA ligase, Q5 High-Fidelity DNA Polymerase, and Gibson assembly kit were purchased from New England Biolabs. Oligonucleotides were synthesized by Genewiz and Sangon Biotech. Oligonucleotide sequences are listed in Supplementary Table 1. Gateway LR reactions and Gibson assembly reactions were performed by following the manufacturer’s protocol. Cell Titer 96 MTS kit for MTS assay was purchased from Promega. Recombinant Human IL-2 was purchased from Peprotech.

### Plasmid construction

Primers were shown in Supplementary Table 1. Adenoviral vector was originally purchased from Clontech and Life Technology, or assembled by using PCR-amplified and synthesized DNA fragments. When required, equal molar amounts of oligonucleotides were annealed in 1 × PNK buffer by heating to 95 °C and gradually cooling down (−1 °C per min) to 37 °C, and then 1 µM of annealed product was phosphorylated by 0.5 units µL^−1^ PNK in presence of 0.5 mM ATP. Gateway LR reactions and Gibson assembly reactions were performed by following manufacturer’s protocol. Golden Gate reactions were performed as described^38^. First, gene parts including the promoter, coding regions and microRNA binding sites were separately cloned into vectors with type IIS restriction sites Esp3I. Second, these vectors were cut by Esp3I and ordered ligated into a vector with type IIS restriction sites BsaI. Last, these circuits were cut by BsaI and ordered ligated into a Gateway vector. The plasmid information was listed in Supplementary Table 2.

### Cell culture and transfection

Cells were cultured in high-glucose Dulbecco’s modified Eagle’s media supplemented with 10% FBS, 4.5 g L^-1^ glucose, 0.045 units mL^−1^ of penicillin and 0.045 g mL^−1^ streptomycin (DMEM complete media) at 37 °C, 100% humidity, and 5% CO_2_, except that Hep3B was cultured in RPMI1640 media plus 10% FBS. One day before transfection, ∼2 × 10^5^ cells in 0.5 mL of high-glucose DMEM complete media were seeded into each well of 24-well plastic plates (Corning). Shortly before transfection, the medium was replaced with fresh DMEM complete media. The transfection experiments were performed as the manufacturer’s protocol by Lipofectamine 3000 transfection reagent or Attractene transfection reagent. pDT7004 (pUBI-linker-NOS), which contains a maize ubiquitin promoter (UBI) followed by a NOS terminator with no protein-coding sequences between UBI and NOS, was used to ensure that the amount of plasmid DNA was equal^26^. The amount of plasmid DNA used in transfection experiments was listed in Supplementary Table 3. Cells were cultured for 2 days before flow cytometry analysis, collection of supernatant containing immune effectors, or puromycin selection.

### Fluorescence-activated cell sorting (FACS) measurement

Cells were trypsinized 48 h after transfection and centrifuged at 300 × *g* for 8 min at 4 °C. The supernatant was removed, and the cells were re-suspended in 1 × PBS that did not contain calcium or magnesium. Fortessa flow cytometer (BD Bio-sciences) were used for FACS analysis, as described^26^, with the following settings: EBFP was measured using a 405 nm laser and a 450/50 filter with a photomultiplier tube (PMT) set at 270 V; EYFP was measured with a 488 nm laser and a 530/30 filter using a PMT set at 210 V; mKate2 was measured with a 561 nm laser and a 670/30 filter using a PMT set at 380 V; iRFP was measured using a 640 nm laser and a 780/60 filter with a PMT set at 480V. For each sample, ∼1 × 10^5^ to ∼5 × 10^5^ cell events were collected.

### RNA extraction and quantitative RT-PCR

RNA was extracted from Chang, HepG2, Huh7, PLC, Hep3B and Hepa1-6 cells using miRNeasy mini kit (Qiagen). The miScript II RT kit (Qiagen) was used for mRNA expression analysis. The real-time PCR reactions were performed in triplicates, using SYBR Select Master Mix (Life Technologies). Relative changes in gene expression were calculated using the 2^−ΔΔCT^ method. The GAPDH expression level was used as a normalization control. The primers used for quantitative RT-PCR were listed in Supplementary Table 1.

### Packaging and titration of OV

Adeno-X 293 cells (purchased from Clontech) were plated at a density of 1 × 10^6^ cells per 60 mm culture plate one day before transfection. After digestion by PacI, 10 μg of linearized OV DNA was transfected into 60 mm culture plate with Lipofectamine 3000 transfection reagent. To collect the VPs, cells were collected one week after transfection, centrifuged at 1500 × *g* for 5 min at room temperature and re-suspended in 500 μL of 1 × PBS. Then cells were lysed with three consecutive ‘freeze-thaw’ cycles. To amplify virus production, the lysate was transferred to a fresh plate with 6 × 10^6^ Adeno-X 293 cells. The virus production can be further amplified by repeating the above procedure to collect more cell lysate. The supernatant containing viruses was concentrated by PEG8000 (Sangon Biotech) to a final volume of 5 mL, and then centrifuged with CsCl gradients (1.4 and 1.2 g mL^−1^) at 100,000 × *g* for 8 h. To measure the virus titer, 5 × 10^5^ Adeno-X 293 cells were seeded in 12-well plate and infected with the 1 μL of purified virus. Cells were collected 6 h after infection, and the total DNA was extracted by using DNeasy Blood & Tissue kit (Qiagen). Quantitative PCR (qPCR) were performed to estimate the virus titer by using qL2 primers shown in Supplementary Table 1.

### Cell proliferation assay after virus infection

Two days before infection, ~10^4^ HepG2 or Huh7 cells in 100 μL of high-glucose DMEM complete medium were seeded into each well of 96-well plastic plates. MTS assay was performed by following the manufacturer’s protocol (Promega) at indicated time point after infection without or with different MOI of viruses. A standard curve was established by measuring MTS readouts of a serial of known amounts of HepG2 or Huh7 cells. The numbers of survival cells after virus infection were estimated using the established standard curve.

### Cytokine production and detection

3 × 10^6^ HepG2 cells were seeded into 12-well plates, infected with 10 MOI of OV. The supernatants were collected at indicated time points, and mouse GM-CSF levels were detected by Mouse GM-CSF ELISA MAX kit (BioLegend) (Fig. 4d). About 2 × 10^5^ of HepG2 and mHepa-6 ce seeded into 12-well plates, infected with 10 MOI of OV. The supernatants were collected at indicated time points, and human GM-CSF levels were detected by Human GM-CSF ELISA MAX kit (BioLegend) (Supplementary Fig. 7b, c). The bioactivity of human IL-2 was analyzed by MTS cell viability assay on the cytokine-dependent cell line CTLL-2. CTLL-2 was purchased from ATCC, and cultured in high-glucose DMEM complete media at 37 °C, 100% humidity, and 5% CO_2_. After washed three times with RPMI1640, 1 × 10^4^ of CTLL-2 cells were seeded into 96-well plates with twofold serial diluted standard IL-2 and measured samples, and incubated for 24 h. MTS assay was performed by following the manufacturer’s protocol (Promega). The standard curve was established by measuring MTS readouts of a serial of known amounts of standard IL-2, and the amounts of IL-2 in measured samples were estimated by using the standard curve.

### Functional assay of anti-PD-1/PD-L1-scFv

3 × 10^6^ HepG2 cells were seeded into 12-well plates, infected with anti-PD-1/PD-L1-scFv encoding OV at MOI of 10. The anti-PD-1/PD-L1-scFv supernatant was collected at indicated time points. The supernatant was added at a dilution of 1:10 into isolated mouse splenocytes that was activated by anti-mouse CD3 antibody (BioLegend) at a concentration of 2 μg mL^−1^. After 48 h of incubation at 37 °C, IFN-γ produced in the media was measured by using Mouse IFN-γ ELISA MAX kit (BioLegend).

### Animal studies

For human HCC xenograft models, six week old female BALB/c nude mice were administered subcutaneous injections with HepG2 (5 × 10^6^ in matrigel) or Huh7 (1 × 10^6^ in PBS) tumor cells. For mouse HCC xenograft model, 2 × 10^6^ Hepa1-6 cells were subcutaneously injected on the right flank of six week old female C57BL/6 mice. Sixty days after the first OV injection, the mice survived after the treatment with oncolytic viruses were re-challenged with 2 × 10^6^ Hepa1-6 tumor cells on the left flank. Tumor size was measured every 3 days and the tumor volume was calculated by using the formula: Volume = 0.5 × length × width^2^. After tumor volume exceeded 100 mm^3^, mice were divided randomly into treatment groups. 1 × 10^9^ VP of indicated adenoviruses or PBS were injected twice into individual tumors at day 0 and day 6. All mice were euthanized when the tumor size exceeded ~1700 mm^3^. All animal experiments were performed in accordance with the National Institute of Health Guide for the Care and Use of Laboratory Animals along with approval from the Institutional Animal Care and Use Committee of Tsinghua University, Beijing, China.

### Histopathology

2 × 10^6^ Hepa1-6 cells were inoculated to six week old C57BL/6 mice subcutaneously. 1 × 10^8^ VP of indicated OVs encoding immunomodulators or control reagent were intratumorally administrated after tumor volume exceeded 100 mm^3^. Specimens from tumor tissues of treated mice were fixed in 4% buffered paraformaldehyde (PFA) and paraffin embedded. Sections of 5-micron thickness were prepared from all specimens, stained with hematoxylin and eosin (H&E) and examined microscopically.

### Analysis of tumor infiltrating lymphocytes

To analyze tumor infiltrating lymphocytes, tumors were isolated from mice, cut into small pieces and digested for 30 min in DMEM media containing 1 mg mL^−1^ collagenase IV (Life Technology), and 50 units mL^−1^ DNase I (Solarbio). Mononuclear cells were isolated using a 40/70% Percoll (GE Healthcare) gradient. For IFN-γ staining, cells were stimulated with 20 ng mL^−1^ PMA (Sigma-Aldrich) and 1 μg mL^−1^ ionomycin (Sigma-Aldrich) in complete RPMI media with 10 μg mL^−1^ brefeldin A (Sigma-Aldrich) at 37 °C for 4 h. Subsequently, cells were stained with antibodies against surface markers, followed by fixation with Fixation/Permeabilization solution (BD Bioscience), IFN-γ was labeled with PE-conjugated anti-mouse IFN-γ antibody at 1:100 dilution (Clone XMG1.2, BD Bioscience) in 1 × Perm/Wash™ Buffer (BD Bioscience). For Ki-67 staining, cells were stained with antibodies against surface markers for 20 min at 4 °C, fixed and permeabilized with a Transcription Factor Staining Buffer Kit (eBioscience), followed by staining with PE-conjugated anti-mouse Ki-67 antibody at 1:100 dilution (Clone 16A8, Biolegend). Brilliant Violet 421 conjugated Anti-CD45.2 antibody at 1:100 dilution  (Clone 104, Biolegend), APC conjugated anti-CD8a at 1:100 dilution (Clone 53-6.7, BD Bioscience) were used for surface markers staining. All cells were analyzed by a flow cytometer and FACS data were processed by using FlowJo (Tree Star) software.

### Bio-distribution of OV

Huh7 tumor-bearing nude mice received a dose of 10^9^ OV intratumorally. Organs and tissue samples were collected and snap frozen in liquid nitrogen at day 7 or day 14 after administration. Adenoviral genome in tissue lysate was extracted using TIANamp Genomic DNA Kit (Tiangen Biotech) after homogenization. Viral titers from individual organ and tissue samples were determined by qPCR (Supplementary Fig. 4). HepG2 tumor-bearing mice received a dose of 10^9^ OV intratumorally. Organs and tissue samples were collected and snap frozen in liquid nitrogen at day 7 after administration. Adenoviral DNA and RNA in tissue lysate were extracted using TIANamp Genomic DNA Kit (Tiangen Biotech) and RNAiso Plus (TaKaR). Viral titers and relative RNA expression level from individual organ and tissue samples were determined by qPCR with qL2 primers and GAPDH primers (Fig. 5c, Supplementary Fig. 2a, b).

### Mathematical modeling of oncolytic virus therapeutic system

We established the mathematic model based on previous studies, describing the interactions among uninfected cancer cells (*S*), infected cancer cells (*I*), free viruses (*V*), cytotoxic lymphocytes recognizing cancer-specific and virus-specific antigen (*Z*_*T*_ and *Z*_*V*_) and immune effector against immunosuppression (*F*)^33,34^. In the model, uninfected cancer cells (*S*) were infected by free viruses (*V*), generating infected cancer cells (*I*). Infected cancer cells were lysed, and meanwhile new viruses were released. Only two types of cytotoxic lymphocytes were considered in the model. One type of lymphocytes (*Z*_*T*_) can be activated by the cancer-specific antigen released from the lysed infected cancer cells (*I*) and then kill cancer cells (both *S* and *I*). The others (*Z*_*V*_) can be activated by the virus-specific antigen and attack cancer cells presenting the virus-specific antigen (*I*). To evaluate the synergistic effect of oncolytic virus and immune modulator, immune effector (*F*) exogenously added or encoded by the virus to relieving the immunosuppression was included in the model.

To calculate the kinetic details, we assumed that most production, degradation and interactions of components in the system followed mass action law. The proliferation of uninfected cancer cells (*S*) followed a logistic growth model, where *γ* indicated the growth rate and *K* indicated the carrying capacity. Infected cancer cells (*I*) lyse at a rate of *δ* and release *α* fold of new viruses. Free viruses (*V*) infected uninfected cancer cells (*S*) at a rate of *κ* and were cleared (by means of infecting normal cells, leaving micro-environment, being neutralized by antibodies or degradation) at a rate of *ω*. Cytotoxic lymphocytes (*Z*_*V*_ and *Z*_*T*_) respectively proliferated at the maximum rate of *c*_*V*_ and *c*_*T*_, killed target cells at the rate of *p*_*V*_ and *p*_*T*_, and were degraded at a rate of *β*. Immune effectors (*F*) were degraded at a rate of *λ*_*F*_ and produced at a rate of *λ*_*I*_ when virus-encoded. A ratio of *ϕ* of cytotoxic lymphocytes were not immunosuppressed, while the rest only proliferated with the aid of immune effectors (*F*). *K*_*F*_ indicated the amount of immune effectors (*F*) to reach half activation.

For simplicity, we made several assumptions. (1) Values changed continuously, which was the limitation of a continuous ODE model. (2) Free viruses (*V*) and uninfected cancer cells (*S*) fully contacted with each other; the distribution and spreading of viruses were not considered. (3) Infected cancer cells did not proliferate, and could not be infected again. (4) Multiple processes in the immune system like antigen presenting were excluded in this model and only two types of representative cytotoxic lymphocytes (*Z*_*V*_ and *Z*_*T*_) were considered. The proliferation rate of cytotoxic lymphocytes recognizing the virus-specific antigen (*Z*_*V*_) was proportional to target cells (*I*). However, the proliferation rate of cytotoxic lymphocytes recognizing the cancer-specific antigen (*Z*_*T*_) followed a second-order reaction with the assumption that the cancer-specific antigens were hidden before lysis of infected cancer cells (*I*). (5) For simplicity, we assumed that immune effectors only functioned to relieve immunosuppression, and immunosuppressed lymphocytes only proliferated with the aid of immune efectors (*F*). The effect of immune effectors (*F*) on boosting proliferation of immunosuppressed cytotoxic lymphocytes was assumed to satisfy a Michaelis-Menten Eq. (6) The ratio of immunosuppression, the impact of immune effectors, the rate of killing target cells and degradation of both cytotoxic lymphocytes were assumed to be equal, and we simulated the proliferation rate of both cytotoxic lymphocytes in a relatively wide range to ensure the generalization and robustness of the observed phenomenon.

The equations for the whole system as shown in Fig. 7a were listed below:

1 $$\frac{{\mathrm{d}S}}{{\mathrm{d}t}} = \gamma S\left( {1 - \frac{{S + I}}{K}} \right) - \kappa SV - p_TSZ_T,$$

2 $$\frac{{\mathrm{d}I}}{{\mathrm{d}t}} = \kappa SV - \delta I - p_VIZ_V - p_TIZ_T,$$

3 $$\frac{{\mathrm{d}V}}{{\mathrm{d}t}} = \alpha \delta I - \omega V - \kappa SV,$$

4 $$\frac{{\mathrm{d}Z_V}}{{\mathrm{d}t}} = c_VIZ_V\left[ {\phi + \frac{{\left( {1 - \phi } \right)F}}{{F + K_F}}} \right] - \beta Z_V,$$

5 $$\frac{{\mathrm{d}Z_T}}{{\mathrm{d}t}} = c_TI\left( {S + I} \right)Z_T\left[ {\phi + \frac{{\left( {1 - \phi } \right)F}}{{F + K_F}}} \right] - \beta Z_T,$$

6 $$\frac{{\mathrm{d}F}}{{\mathrm{d}t}} = \lambda _II - \lambda _FF.$$

The minimal system of oncolytic virus therapeutic system only consisted of uninfected and infected cancer cells, and free viruses (Supplementary Fig. 9a). The model was deduced as follows:

7 $$\frac{{\mathrm{d}S}}{{\mathrm{d}t}} = \gamma S\left( {1 - \frac{{S + I}}{K}} \right) - \kappa SV,$$

8 $$\frac{{\mathrm{d}I}}{{\mathrm{d}t}} = \kappa SV - \delta I,$$

9 $$\frac{{\mathrm{d}V}}{{\mathrm{d}t}} = \alpha \delta I - \omega V - \kappa SV.$$

In the oncolytic virus extended system shown in Supplementary Fig. 10a, cytotoxic lymphocytes were included and assumed not to be immunosuppressed. The extended model was listed as follows:

10 $$\frac{{\mathrm{d}S}}{{\mathrm{d}t}} = \gamma S\left( {1 - \frac{{S + I}}{K}} \right) - \kappa SV - p_TSZ_T,$$

11 $$\frac{{\mathrm{d}I}}{{\mathrm{d}t}} = \kappa SV - \delta I - p_VIZ_V - p_TIZ_T,$$

12 $$\frac{{\mathrm{d}V}}{{\mathrm{d}t}} = \alpha \delta I - \omega V - \kappa SV,$$

13 $$\frac{{\mathrm{d}Z_V}}{{\mathrm{d}t}} = c_VIZ_V - \beta Z_V,$$

14 $$\frac{{\mathrm{d}Z_T}}{{\mathrm{d}t}} = c_TI\left( {S + I} \right)Z_T - \beta Z_T.$$

Half killing time and maximum tumor size before half killing were calculated to evaluate simulated therapeutic effects. The initial tumor size was set to 1. Half killing time (HT) was defined as the time when the tumor size was first decreased to 0.5 within 100 a.u. of simulation time, and the maximum tumor size before half killing (MS) was also recorded as the indication of therapeutic efficacy.

Response score and regression score were calculated to evaluate simulated therapeutic effects among varying strength of lymphocyte responses against cancer-specific and virus-specific antigen, and higher scores indicated higher robustness of therapeutic efficacy. Response score (RP) was defined as the normalized sum of reduced HT, and regression score (RG) was defined as the normalized sum of reduced MS:

15 $${\mathrm{RP}} = \mathop {\sum}\nolimits_{ij} {\left( {1 - \frac{{{\mathrm{HT}}_{{\mathrm{ij}}}}}{{{\mathrm{HT}}_{{\mathrm{max}}}}}} \right)}$$

16 $${\mathrm{RG}} = \mathop {\sum}\nolimits_{ij} {\left( {1 - \frac{{{\mathrm{MS}}_{{\mathrm{ij}}}}}{{{\mathrm{MS}}_{{\mathrm{max}}}}}} \right)}$$

In the above formulas: i and j indicated each strength of lymphocyte responses against cancer-specific and virus-specific antigen. HT_max_ indicated the duration of simulation. MS_max_ indicated the carrying capacity.

To measure the degradation rate of oncolytic virus in vitro, indicated amounts of viruses in 100 μL of high-glucose DMEM complete media were placed in 96-well plate incubated at 37 °C for either 0 day or 8 days. The quantity of viral particles was measured by using the qPCR method as mentioned. Virus degradation rate (*ω*) was estimated by fitting the data with an exponential degradation model.

When fitting the minimal model to measured numbers of HepG2 or Huh7 cells infected with different amounts of viruses up to 5 days (Supplementary Fig. 9c), we assumed that *K* was infinity. Therefore, the logistic growth model of uninfected cancer cells (*S*) was simplified as an exponential growth model. We also assumed that *ω* is equal to 0 as estimated by in vitro results shown in Supplementary Fig. 9b. Simplified model was shown as follows.

17 $$\frac{{\mathrm{d}S}}{{\mathrm{d}t}} = \gamma S - \kappa SV,$$

18 $$\frac{{\mathrm{d}I}}{{\mathrm{d}t}} = \kappa SV - \delta I,$$

19 $$\frac{{\mathrm{d}V}}{{\mathrm{d}t}} = \alpha \delta I - \kappa SV.$$

The remaining parameters in this model were fitted from the experimental data as shown in Supplementary Fig. 9c by using the stepwise least-squares regression. Briefly, we fitted the data obtained in the absence of viruses with an exponential growth model to estimate the proliferation rate *γ* and initial cancer cell numbers. The infection rate *κ*, the lysing rate *δ* and the viral replication number *α* were fitted by using the lsqcurvefit function in MATLAB. We tested the algorithm with multiple initial values to avoid trapping into local solutions. All the parameters used in the model were listed in Supplementary Table 4.

### Reporting summary

Further information on research design is available in the Nature Research Reporting Summary linked to this article.

## Supplementary information

Supplementary Information

Reporting Summary

Source Data

## Data Availability

The authors declare that all data supporting the findings of this study are available within the paper and its Supplementary Information files or upon reasonable request. All plasmids were submitted to GE Share (https://www.geshare.com.cn) with ID 1774 ~ 1829. Source data for the figures are available in the Source Data file.
