# Supplementary material for: Oncolytic adenovirus programmed by synthetic gene circuit for cancer immunotherapy
Source: Nat Commun. 2019 Oct 22;10:4801. doi: 10.1038/s41467-019-12794-2 (PMC6805884; doi:10.1038/s41467-019-12794-2)
Supplement: Supplementary file 1 — Supplementary Information [file 41467_2019_12794_MOESM1_ESM.pdf]

Supplementary Information

Oncolytic adenovirus programmed by synthetic gene circuit for cancer immunotherapy

Huang et al.,

*Correspondence: zhenxie@tsinghua.edu.cn

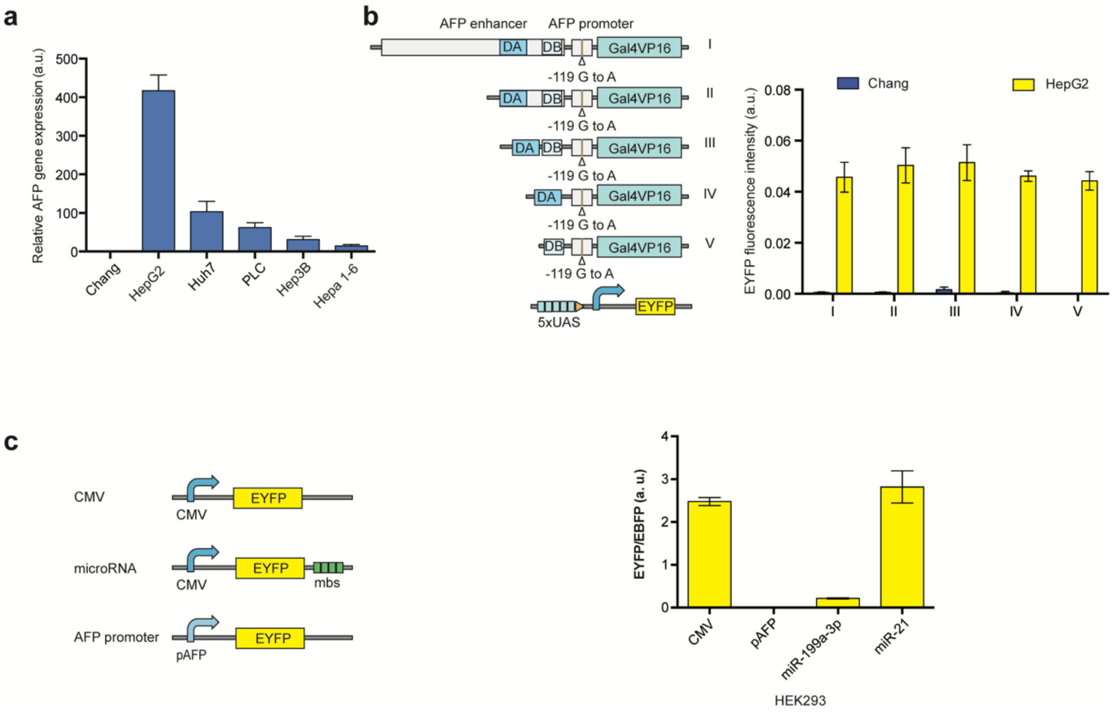

Supplementary Fig. 1 The biomarker background of different cell lines. **a** Analysis of AFP expression level in different cell lines. Quantitative RT-PCR results were shown as mean ± s.d. from three independent replicates. **b** Characterization of AFP promoter (I) and its derivatives (from II to V) in Chang and HepG2 cells by transient transfection. DA and DB, domain A and B in AFP enhancer; arrow points to a G-to-A mutation in AFP promoter. **c** Assay of the AFP promoter activity and microRNA expression levels in HEK293 cells. CMV, the CMV-driven EYFP plasmid; microRNA, the CMV-driven EYFP with indicated miRNA binding sites in the 3’-UTR; AFP promoter, the AFP-driven EYFP plasmid. Data are shown as mean ± s.d. from three independent replicates. Source data are provided as a Source Data file.

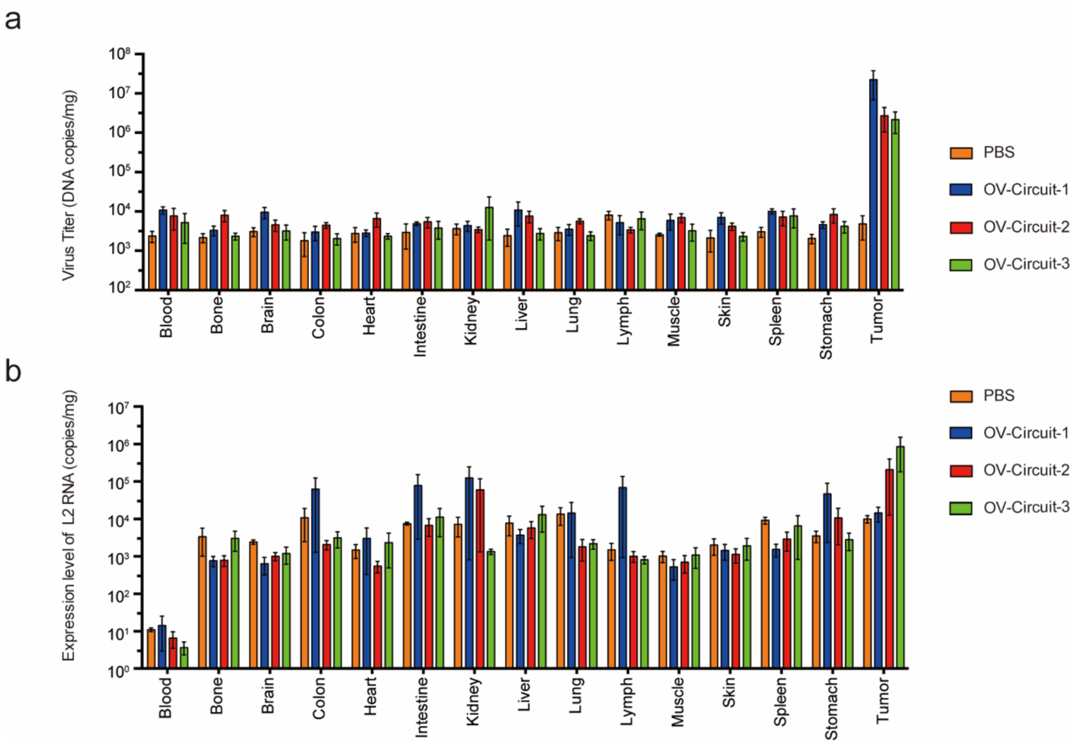

Supplementary Fig. 2 Distribution of virus under the control of different circuit switches **a** DNA distribution of OV-Circuits in HepG2 xenografted nude mice 3 days after injection of 1 × 10^9 VP of OV-Circuits when the size of tumor exceeded ~500 mm^3. Data are shown as mean ± s.d. from three independent replicates by using quantitative PCR. **b** RNA distribution of OV-Circuits in HepG2 xenografted nude mice 3 days after injection of 1 × 10^9 VP of OV-Circuits when the size of tumor exceeded ~500 mm^3. Data are shown as mean ± s.d. from three independent replicates by using quantitative PCR. Source data are provided as a Source Data file.

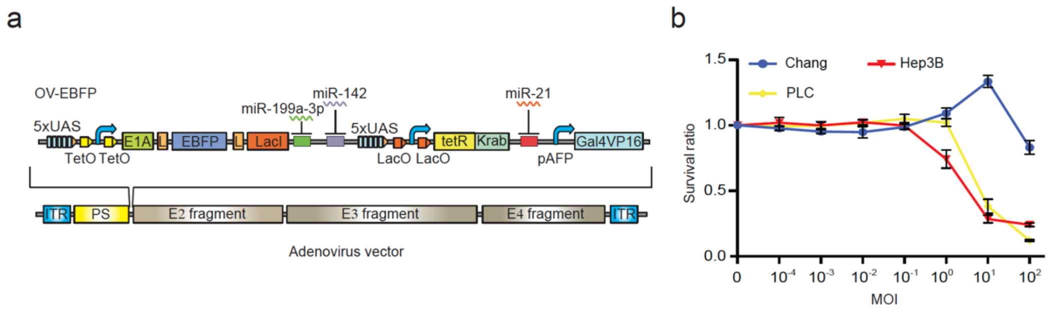

Supplementary Fig. 3 Functional assay of OV-EBFP in HCC cell lines. **a** The schematic diagram of OV-EBFP. UAS, upstream activation sites; tetO, binding site of tetR:Krab repressor; LacO, binding site of LacI repressor; L, self-cleavage 2A linker; pAFP, AFP promoter derivative III. **b** The survival ratios of PLC and Hep3B cells were measured by using MTS cell viability assay 6 days after incubation with indicated amount of OV-EBFP. Data are shown as mean ± s.d. from one experiment representative of three independent experiments with similar results. Source data are provided as a Source Data file.

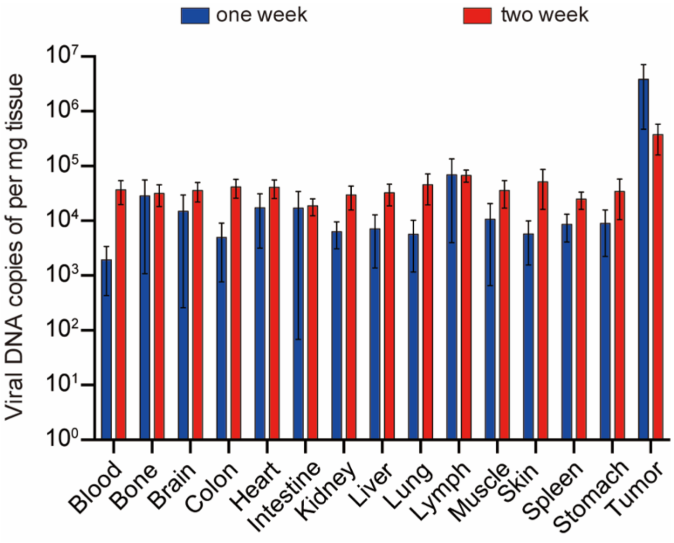

Supplementary Fig. 4 Distribution of OV-EBFP DNA in Huh7 xenografted nude mice 1 or 2 week(s) after injection of 1×10^9 VP of OV-EBFP when the size of tumor exceeded ~500 mm^3. Data are shown as mean ± s.d. (n=6 or 7). Source data are provided as a Source Data file.

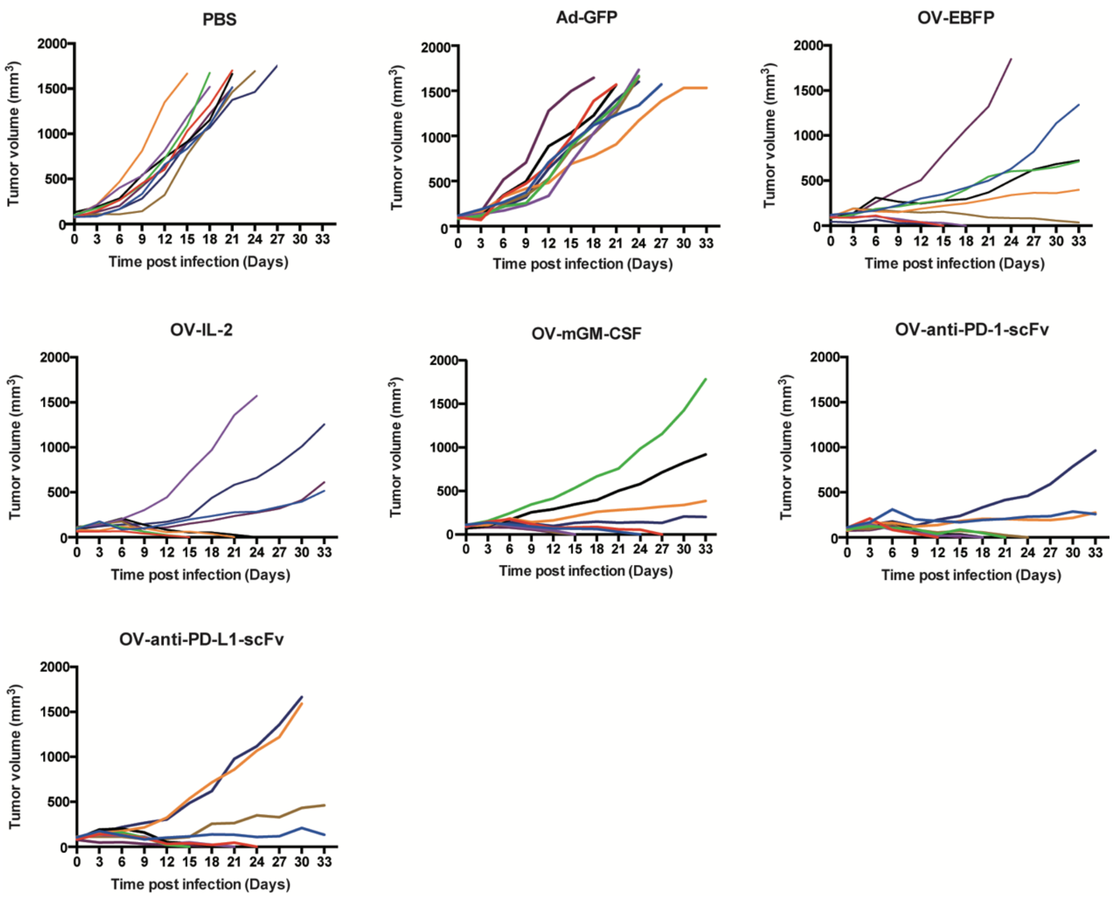

Supplementary Fig. 5 Effect of synthetic oncolytic adenovirus in immune-competent Hepa 1-6 mouse models. Each mouse was intratumorally injected with 1×10^9 VP of indicated OV-Effector or Ad-GFP twice in one week right after the size of Hepa1-6 tumor reach to 100 mm^3. PBS was used as a negative control. Tumor growth curve are shown (n=9 or 10). Source data are provided as a Source Data file.

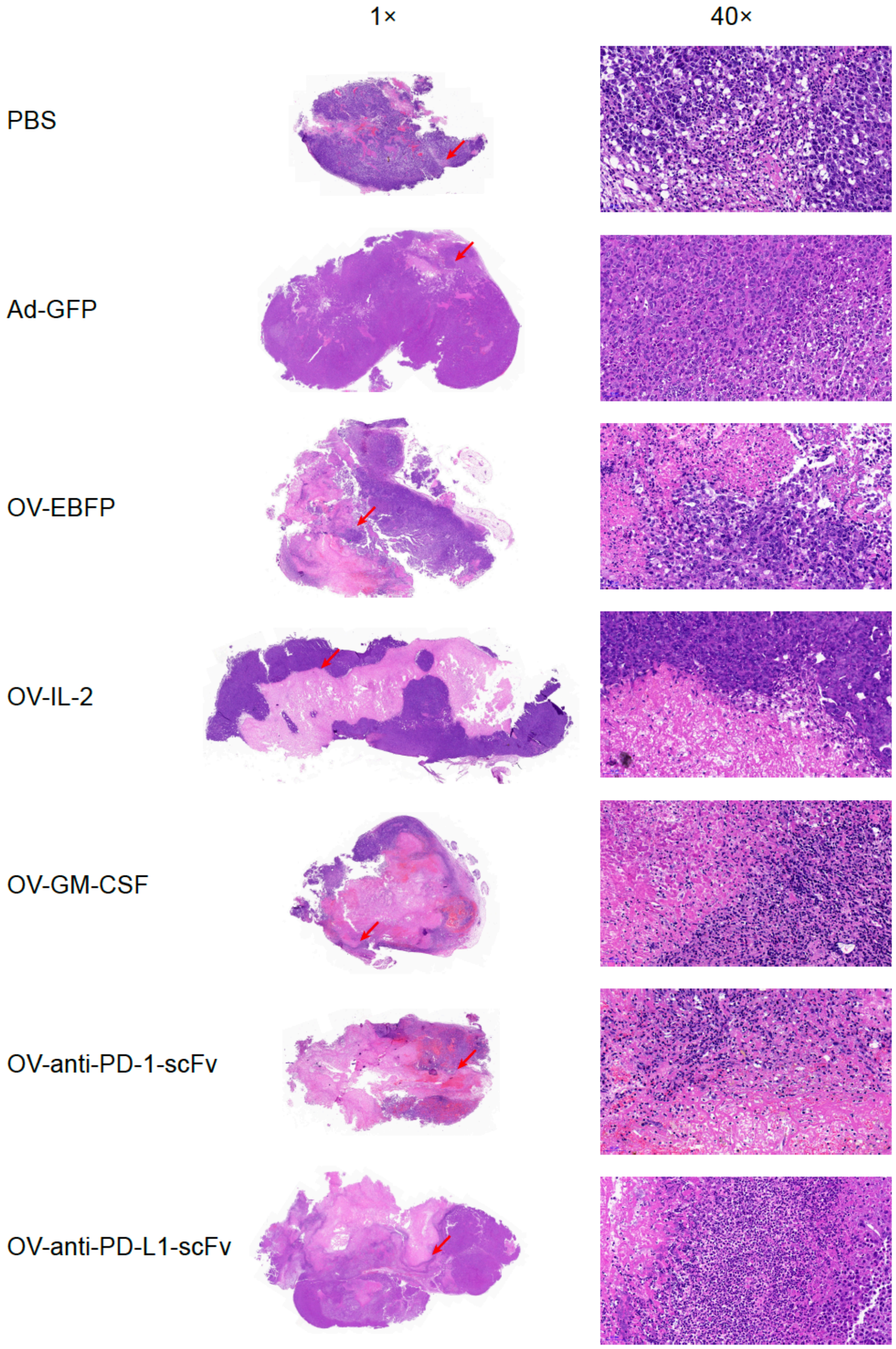

Supplementary Fig. 6 Histopathology of treated Hepa1-6 tumors in xenografted C57BL/6 mice. Hepa1-6 tumor bearing mice were treated with indicated oncolytic adenovirus or PBS control agent for 14 days. Tumor sections of 5-micron thickness

were stained with hematoxyline and eosin (H&E) and examined microscopically. The red arrows in microscopic images with 1 × amplification indicate the position of microscopic images with 40 × amplification.

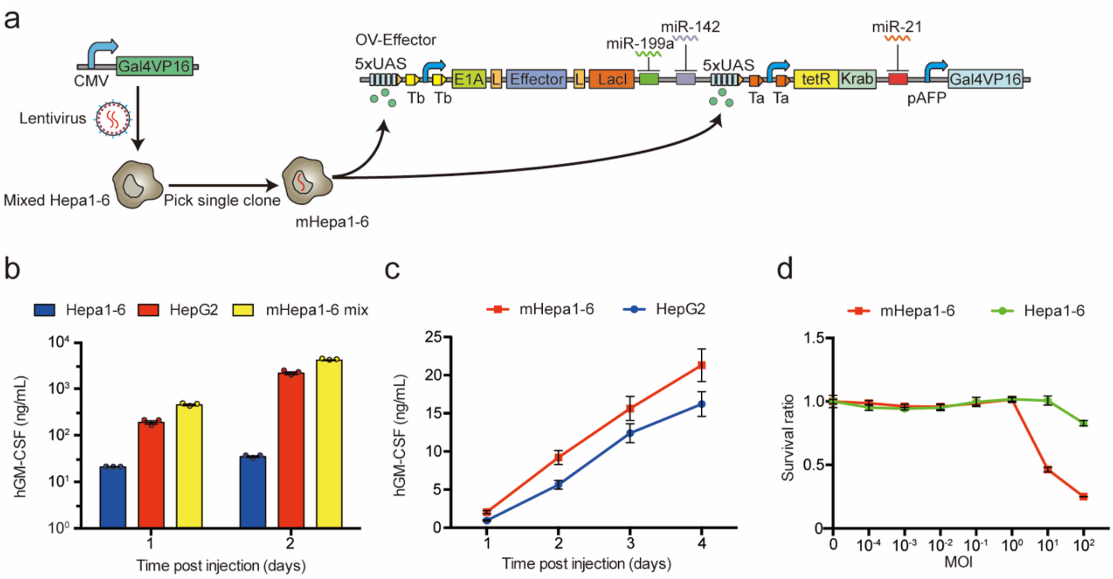

Supplementary Fig. 7 Construction of modified Hepa1-6 (mHepa1-6). **a** The schematic diagram of mHepa1-6 construction process. **b** 3 × 10^6 Hepa1-6, HepG2 and mHepa1-6 mix cells were incubated with 10 MOI of oncolytic adenovirus encoding human GM-CSF (hGM-CSF) for 48 hours and the supernatants were collected for measurement of hGM-CSF production using ELISA. **c** 3 × 10^6 mHepa1-6 cells and HepG2 cells were incubated with 10 MOI of oncolytic adenovirus encoding human GM-CSF (hGM-CSF) for 4 days and the supernatants were collected for measurement of hGM-CSF production using ELISA. **d** The survival ratios of mHepa1-6 and Hepa1-6 cells were measured by using MTS cell viability assay 6 days after incubation with indicated amount of OV-EBFP. Data are shown as mean ± s.d. from one experiment representative of three independent experiments with similar results. Source data are provided as a Source Data file.

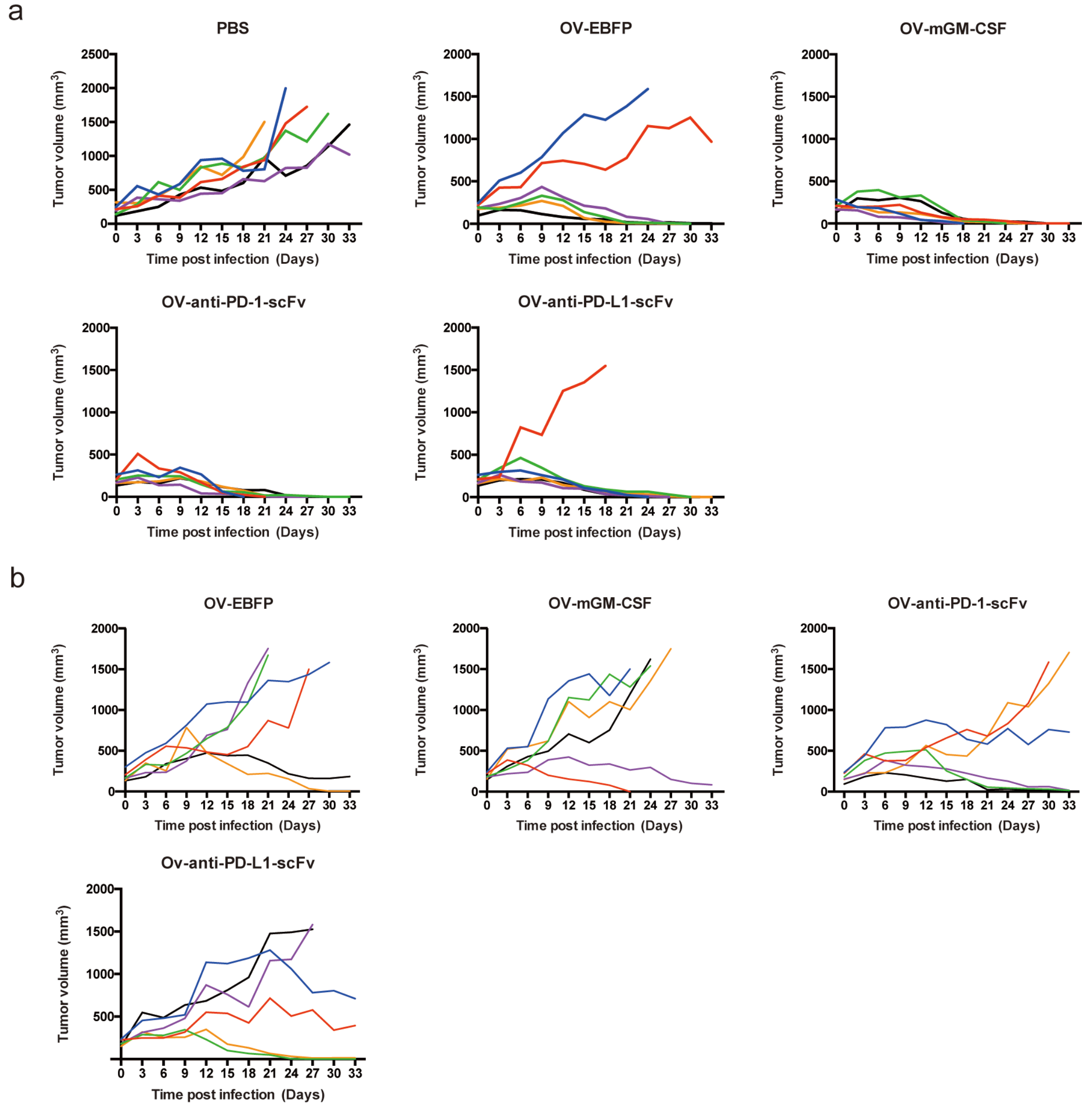

Supplementary Fig. 8 Effect of synthetic oncolytic adenovirus in immune-competent mHepa 1-6 mouse models. **a** Each mouse was intratumorally injected with 1×10^9 VP of indicated OV-Effector twice in one week right after the size of mHepa1-6 tumor reach to 100 mm^3. PBS was used as a negative control. Tumor growth curve are shown (n=6). **b** Each mouse was intratumorally injected with 1×10^7 VP of indicated OV-Effector twice in one week right after the size of mHepa1-6 tumor reach to 100 mm^3. PBS used as a negative control was the same group in **a**. Tumor growth curve are shown (n=6). Source data are provided as a Source Data file.

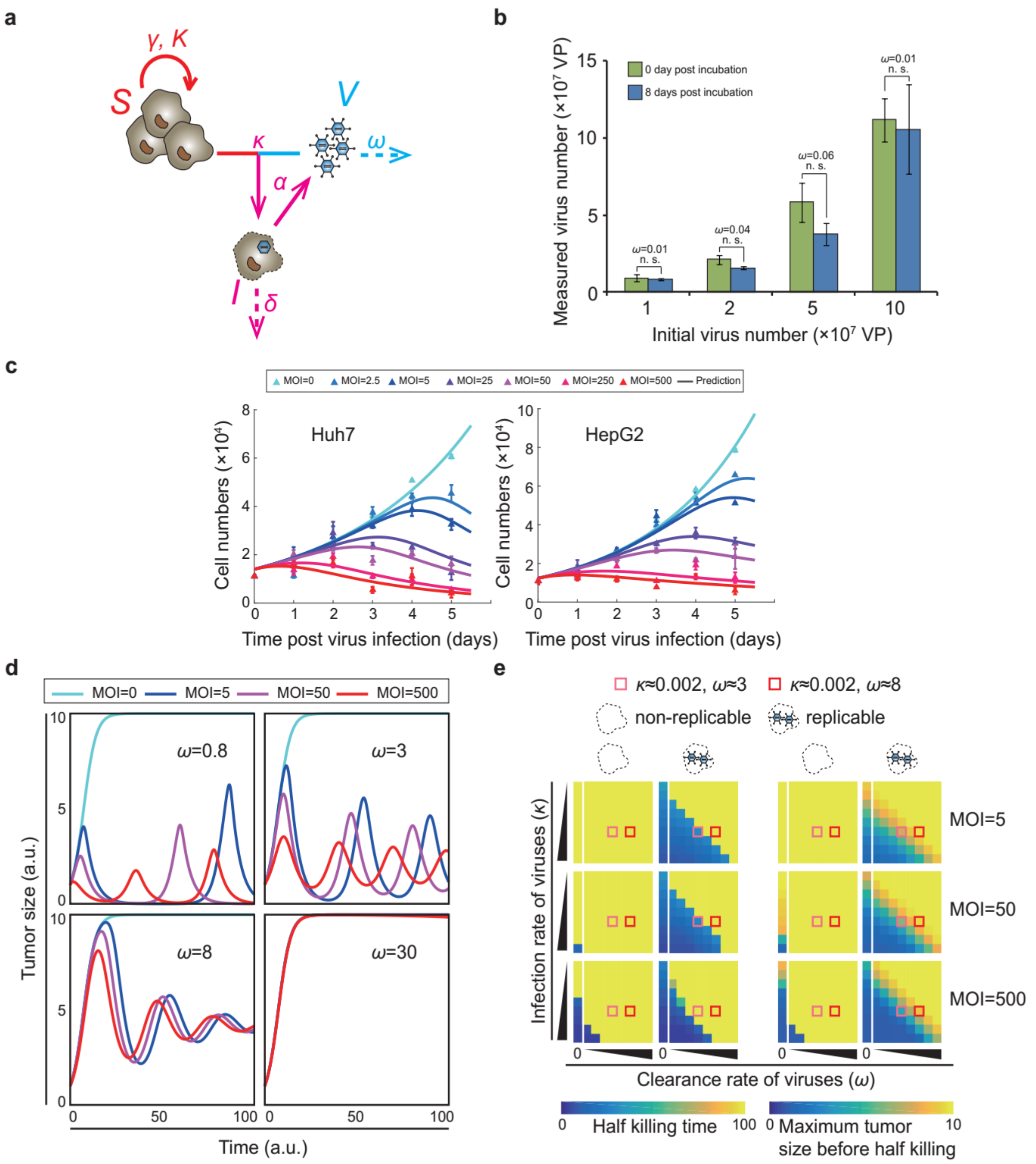

Supplementary Fig. 9 The minimal model of oncolytic virus system. **a** Schematic diagram of the minimal model of oncolytic virus system consisting of uninfected cancer cells (*S*), infected cancer cells (*I*) and free viruses (*V*). All parameters are listed in Supplementary Table 4. **b** Measurement of adenovirus degradation rate *in vitro*. Indicated amount of OV-EBFP was incubated in 100 μL complete DMEM media for 8 days, and the numbers of OV-EBFP before or after incubation was measured by using quantitative PCR to estimate virus degradation rate (ω). Student's t-test was performed: n. s., not significant ($P>0.05$). **c** HepG2 and Huh7 cells were incubated with indicated amount of OV-EBFP, and MTS cell viability assay was used to estimate the number of survived cells at indicated time point (See details in **Methods**). Each data point shows

mean ± s.d. from three independent replicates. Solid lines indicate the predicted growth curves by fitting to the minimal model shown in Supplementary Fig. 9a. Fitted parameters were listed in **Methods**. **d** Simulated tumor growth curves by using the minimal model with varying MOI and virus clearance rates (ω). **e** Half killing time and maximum tumor size before half killing (see details in **Methods**) was calculated based on the minimal model with varying MOI. Each matrix shows parameter combinations of virus infection rate κ (10^{-4}~10^{-2} on the logarithmic scale) and virus clearance rate ω (0~30 on the logarithmic scale). Dash circle, non-replicable virus (α=0); dash circle with 2 virus symbols, replicable virus (α=1800). Pink squares (κ≈0.002, ω≈3) and red squares (κ≈0.002, ω≈8) indicate the same parameter combinations marked in Supplementary Fig. 10. Source data are provided as a Source Data file.

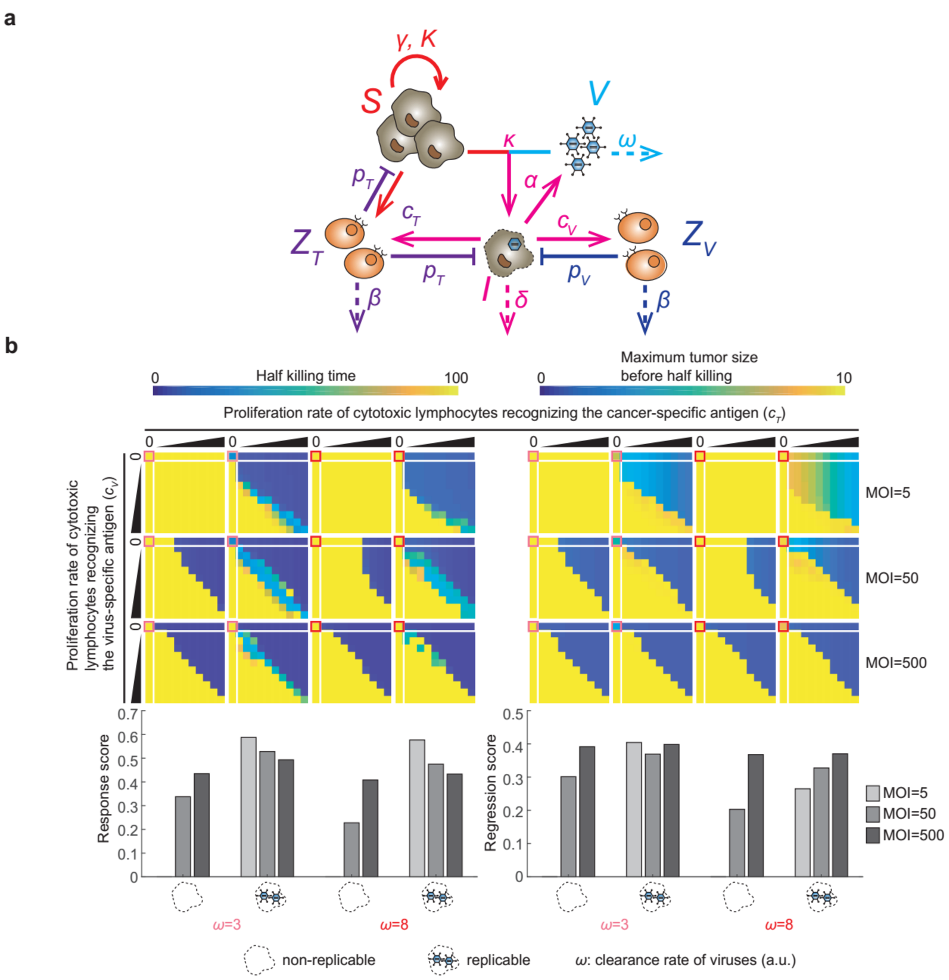

Supplementary Fig. 10 The model of oncolytic virus therapeutic system with cytotoxic lymphocytes. **a** Schematic diagram of the model of oncolytic virus therapeutic system consisting of uninfected cancer cells (S), infected cancer cells (I), free viruses (V), cytotoxic lymphocytes recognizing virus-specific antigen (Z_V) and cytotoxic lymphocytes recognizing cancer-specific antigen (Z_T). The virus infection rate (κ=0.002); all other parameters are listed in Supplementary Table 4. **b** Half killing time, maximum tumor size before half killing, response score and regression score (see details in **Methods**) was calculated based on the model in panel **a** with varying MOI and virus clearance rates. Each matrix shows parameter combinations of proliferation rate of cytotoxic lymphocytes recognizing virus-specific antigen (c_V, 0 and $10^{1.6}$~$10^{2.8}$ a.u.) and cancer-specific antigen (c_T, 0 and $10^{1.2}$~$10^{2.4}$ a.u.). Dash circle, non-replicable virus (α=0); dash circle with 2 virus symbols, replicable virus (α=1800). Pink squares

(κ=0.002, ω=3) and red squares (κ=0.002, ω=8) indicate the same parameter combinations marked in Supplementary Fig. 9.

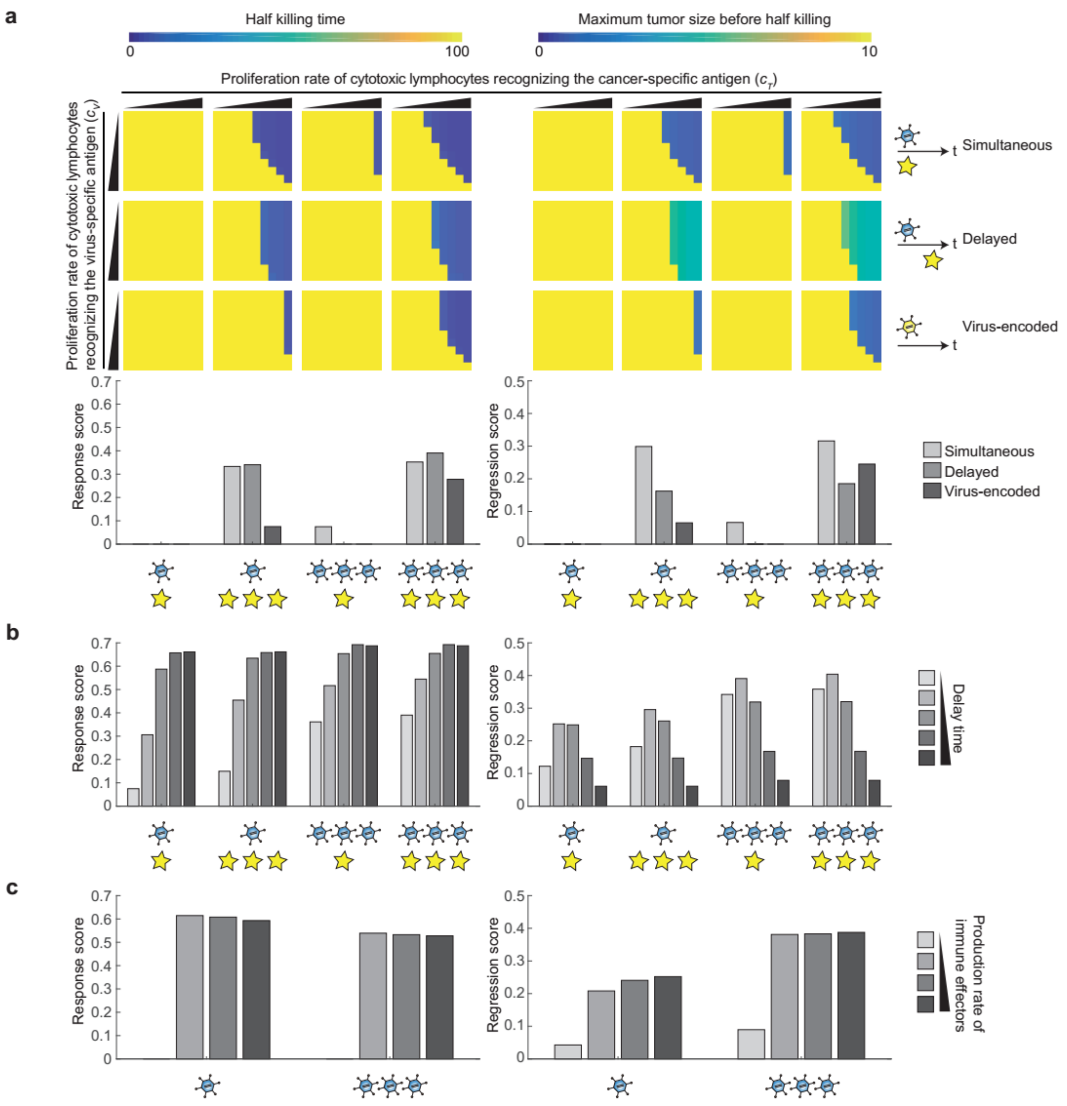

Supplementary Fig. 11 Analysis of virus replicability and immune effector administration in oncolytic virus therapeutic system. **a** Predicted therapeutic efficacy of different administration strategies of non-replicable virus and immune effector by using the model shown in Fig. 7a, where immune effector was administrated along with or after the virus injection, or encoded by the virus. Each matrix shows parameter combinations of c_V ($10^{1.6}$~$10^{3.5}$ a.u.) and c_T ($10^{1.2}$~$10^{3.1}$ a.u.). **b** Predicted therapeutic efficacy of replicable virus and delayed immune effector administration with different delay time by using the model shown in Fig. 7a. **c** Predicted therapeutic efficacy of replicable virus and virus-encoded immune effector with different production rate by using the model shown in Fig. 7a. The initial amount of virus (MOI = 5 (1 virus symbol) or 50 (3 virus symbols)) and initial amount or production rate of immune effectors (10

a.u. (1 star symbol) or 50 a.u. (3 star symbols)) are noted on the bottom. Calculation of half killing time, maximum tumor size before half killing, response score and progression score is explained in **Methods**.

Supplementary Table 1. Primers used in this study.

Name	Sequence
qAFP for	TTTGCCCAGTTTGTTC
qAFP rev	CACTTTGGCTGCAGCAG
qL2 for	TTGTGGTTCTTGCAGATATGGC
qL2 rev	TCGGAATCCCGGCACC
GAPDH for	AGAAGGCTGGGGCTCATTTG
GAPDH rev	AGGGGCCATCCACAGTCTTC
AFP promoter for	AAATCTAGAAGTTTGAGGAGAATATTTG
AFP promoter rev	AAAGGATCCTTGCTAGTTATTTTGTTATTG
AFP enhancer for	AAAAGATCTTTAGAAATATGG
AFP enhancer rev	TTTTCTAGAGGCCTGGATAAAGCTG
AFP DA for	AAAAGATCTCAGATTGAATTATTTGCCTGTCATAC
AFP DA rev	AAATCTAGAAAGCTTTTGTAGCCATGTGAGTGG
AFP DB for	AAAAGATCTAAGCTTCCTGATTAATAATTACAC
AFP DB rev-2	AAATCTAGATAGGAAGTTTTCGCAATAATACTGTCAAATAAGTGGCCTGGAT AAAGCTGAGTGG
AFP short enhancer for	AAAAGATCTCAGATTGAATTATTTGCCTGTCATAC
AFP short enhancer rev	AAATCTAGATAGGAAGTTTTCGCAATAATACTGTCAAATAAGTGGCCTGGAT AAAGCTGAGTGG
AFP point mutation for	CAACCTAAGGAAATACCATAAAG
AFP point mutation rev	TTTCCTTAGGTTGAAAATGGGACATTTGTCAATAATTAACAGCATAGCGG
miR-142-1	CAGTAGTGCTTTCTACTTTATGAGTAGTGCTTTCTACTTTATGAGTA
miR-142-2	CATAAAGTAGAAAGCACTACTCATAAAGTAGAAAGCACTACTGGTAC
miR-142-3	GTGCTTTCTACTTTATGAGTAGTGCTTTCTACTTTATGT
miR-142-4	CTAGACATAAAGTAGAAAGCACTACTCATAAAGTAGAAAGCACTACT
miR-199a-3p-1	AGCTTCACAAGATCGGATCTACGGGTTCACAAGATCGGATCTACGGGTT
miR-199a-3p-2	GATATCAACCCGTAGATCCGATCTTGTGAACCCGTAGATCCGATCTTGTGA
miR-199a-3p-3	GATATCCACAAGATCGGATCTACGGGTTCACAAGATCGGATCTACGGGTTG
miR-199a-3p-4	GATCCAACCCGTAGATCCGATCTTGTGAACCCGTAGATCCGATCTTGTG
miR-122-1	AGCTTCAAACACCATTGTCACACTCCACAAACACCATTGTCACACTCCA
miR-122-2	GTTTGTGGAGTGTGACAATGGTGTTTGTGGAGTGTGACAATGGTGTTTGA

miR-122-3	CAAACACCATTGTCACACTCCACAAACACCATTGTCACACTCCAG
miR-122-4	TCGACTGGAGTGTGACAATGGTGTTTGTGGAGTGTGACAATGGT
miR-21-1	AGCTTTCAACATCAGTCTGATAAGCTATCAACATCAGTCTGATAAGCTA
miR-21-2	TTGATAGCTTATCAGACTGATGTTGATAGCTTATCAGACTGATGTTGAA
miR-21-3	TCAACATCAGTCTGATAAGCTATCAACATCAGTCTGATAAGCTAG
miR-21-4	TCGACTAGCTTATCAGACTGATGTTGATAGCTTATCAGACTGATG
FF5-1	AGCTTAAGCACTCTGATTTGACAATTAAAGCACTCTGATTTGACAATTAA
FF5-2	CTAGTAAGCACTCTGATTTGACAATTAAAGCACTCTGATTTGACAATTAG
FF5-3	CTAGTTAATTGTCAAATCAGAGTGCTTTAATTGTCAAATCAGAGTGCTTA
FF5-4	TCGACTAATTGTCAAATCAGAGTGCTTTAATTGTCAAATCAGAGTGCTTA
H160105	AAAGGTCTCACCGGTATGGCTAGATTAGATAAAAG
H160106	AAAGGTCTCGTCGACCTTTCTCTTCTTTTTGGCGACCCACTTTCACATTTAAG
H160107	AAAGGTCTCGTCGACGGCGGTGG
H160108	AAAGGTCTCGGATCCTTAAACTGATGATTTGATTTC
H160109	AAAGGTCTCGAATTCGCCACCATGGCTAGATTAG
H160430	TTTCGTCTCGCTATTTAATTAAGTAACTATAACGGTCGCTCCGAATTTCTCGAG
H160431	AAACGTCTCGGTGGCGGTCGAATTC
H160433	AAACGTCTCATTTCTTAACTAGTATTGTCGAC
H160434	AAACGTCTCGGAAATCGCCAAGCTACGGG
H160437	AAACGTCTCCTTGTATTAAACTGATGATTTGATTTC
H160440	AAACGTCTCATAATCAGCCATACC
H160441	AAACGTCTCCGAGCTGCAGTGAAAAAAATG
H160448	AAACGTCTCAGCTCCAGATTGAATTATTTGC
H160449	AAACGTCTCGGATCCTTGCTAGTTATTTTG
H160450	AAACGTCTCGGATCCGGCCACCATGGCCCCCCCGAC
H160451	AAACGTCTCAGTGAATTATACAGTCAACTGTC
H160452	AAACGTCTCTTCACTCCTCAGGTG
H160453	AAACGTCTCATCGACTTAATTAAATTACCTCTTTCTCCGATCTCCATAAGAGAAGAGG
H150509	ATGAGACATATTATCTGCCACG
H150510	AATGTGGTATGGCTGATTATTTAACTAGTATTGTCGAC
H150511	ATAATCAGCCATACCACATT
H150512	AAAGGTCTCGAATCGCAGTGAAAAAAATG
H160307	AAAGGTCTCGCTCATATGAGACATATTATCTGCC
H160308	AAAGGTCTCCTGTTTGAGGAGGGAAAAATTTGTAGCTCCGGACCCCTCGAGTGGCCTGG
H160309	AAAGGTCTCAAACAGGCTGGAGATGTCGAAGAAAATCCTGGGCCTACCGGTCCCATGGTG
H160313	AAAGGTCTCGGCCGCTTAAACCTTCCTCTTCTTC
H160314	AAAGGTCTCGCGGCCGCTAAC

H160315	AAAGGTCTCCTAGTTTTAACTAGTATTGTCGAC
H150503	AAAACCAAAAGATCTCCGCTGACTAGGG
H150504	CCCTAGTCAGCGGAGATCTTTTGGTTTTGGG
H160420	CTGCCGCGGCCCTCAGATCTCTTGATCTCCACCTTG
H160421	CAAGGTGGAGATCAAGAGATCTGAGGGCCGCGGCAG
H160715	CAGGTCGACGGTCTCATCGAACCCAGCTTTCTTGTACAAAGTTGG
H160716	TGGCTCGAGCGTCTCCAAATAATG
In-Fusion for	AAAACATGTGTAACTATAACGGTCTAGGGATAACAGGGTAATTCTTTCCTGCGTTATCCCC
In-Fusion rev	TTTACGCGTATTACCTCTTTCTCCATTACCCTGTTATCCCTATAAGATACATTGATGAGTTTG

Supplementary Table 2. Plasmid constructs used in this study.

**pH291 [pENTR_L4-AFP enhancer-point mutation -199 (G to A) AFP promoter-R1]** was prepared by inserting the point mutation AFP promoter into pH089 [pENTR_L4-AFP enhancer-T14(+)-point mutation -119 (G to A) AFP promoter-3 × T14-R1] with BamHI and XbaI. The point mutation AFP promoter is made by overlap PCR from HepG2 DNA with AFP promoter for, AFP promoter rev, AFP point mutation for and AFP point mutation rev.
**pH356 (pZD_Seq1-AFP Enhancer-AFP promoter-Gal4VP16-Seq2)** was prepared by combining pH291 [pENTR_L4-AFP enhancer-point mutation -199 (G to A) AFP promoter-R1] and pCH152 (pENTR_L1-Kozak-Gal4VP16-L2) with pZDonor_1-GTW-2 in a Gateway LR reaction.
**pH359 [pENTR_L1-AFP short enhancer -point mutation -199 (G to A) AFP promoter-L4]** was prepared by inserting the short AFP enhancer sequence (821bp) into pH291 [pENTR_L4-AFP enhancer-point mutation -199 (G to A) AFP promoter-R1] with BglII and XbaI. The short enhancer was amplified from HepG2 DNA with AFP short enhancer for and AFP short enhancer rev by PCR.
**pH360 [pZD_Seq1-AFP short enhancer-point mutation -199 (G to A) AFP promoter-Gal4VP16-Seq2]** was prepared by combining pH359 (pENTR_L1-AFP short enhancer -point mutation -199 (G to A) AFP promoter-L4) and pCH152 (pENTR_L1-Kozak sequence-Gal4VP16-L2) with pZDonor_1-GTW-2 in a Gateway LR reaction.
**pH357 [pENTR_L4-AFP enhancer domain A-AFP enhancer domain B-point mutation -199 (G to A) AFP promoter-L1]** was prepared by inserting AFP enhancer domain A enhancer domain B fragment into pH291 [pENTR_L4-AFP enhancer-point mutation -199 (G to A) AFP promoter-R1] with BglII and XbaI. AFP enhancer domain A was amplified from HepG2 DNA with AFP DA for and AFP DA rev-1 by PCR. And domain B was amplified from HepG2 DNA with AFP DB for and AFP DB rev. AFP enhancer domain A enhancer domain B fragment was amplified from these tow fragments with AFP DA for and AFP DA rev by overlap PCR.
**pH358 [pZD_Seq1-AFP enhancer domain A-AFP enhancer domain B-point mutation -199 (G to A) promoter-Gal4VP16-Seq2]** was prepared by combining pH357 [pENTR_L4-AFP enhancer domain A-AFP enhancer domain B-point mutation -199 (G to A) AFP promoter-L1] and pCH152 (pENTR_L1-Kozak sequence-Gal4VP16-L2) with pZDonor_1-GTW-2 in a Gateway LR reaction.

**pH367 [pENTR_L4-AFP enhancer domain A-point mutation -199 (G to A) AFP promoter-R1]** was prepared by inserting AFP enhancer domain A into pH291 [pENTR_L4-AFP enhancer-point mutation -199 (G to A) AFP promoter-R1] with BglII and XbaI. The AFP enhancer domain A was amplified from HepG2 DNA with AFP DA for and AFP DA rev by PCR.
**pH369 [pZD_Seq1-AFP enhancer domain A-point mutation -199 (G to A) AFP promoter-Gal4VP16-Seq2]** was prepared by combining pH367 [pENTR_L4-AFP enhancer domain A-point mutation -199 (G to A) AFP promoter-R1] and pCH152 (pENTR_L1-Kozak sequence-Gal4VP16-L2) with pZDonor_1-GTW-2 in a Gateway LR reaction.
**pH372 [pENTR_L4-AFP enhancer domain B-point mutation -199 (G to A) AFP promoter-R1]** was prepared by inserting AFP enhancer domain B into pH291 [pENTR_L4-AFP enhancer-point mutation -199 (G to A) AFP promoter-R1] with BglII and XbaI. The AFP enhancer domain B was amplified from HepG2 DNA with AFP DB for and AFP DB rev by PCR.
**pH373 [pZD_Seq1-AFP enhancer domain B-point mutation -199 (G to A) AFP promoter-Gal4VP16-Seq2]** was prepared by combining pH372 [pENTR_L4-AFP enhancer domain B-point mutation -199 (G to A) AFP promoter_R1] and pCH152 (pENTR_L1-Kozak sequence-Gal4VP16_L2) with pZDonor_1-GTW-2 in a Gateway LR reaction.
**pH081 [pENTR_L4-T14(+)-AFP promoter-T14 × 3-R1]** was prepared by inserting AFP promoter sequence into pENTR_L4-T14(+) T14(+) × 3-72bp-R1 with EcoRI and XhoI.
**pH089 [pENTR_L4-AFP enhancer-T14(+)-AFP promoter-3 × T14-R1]** was prepared by inserting AFP enhancer sequence into pH081 [pENTR_L4-T14(+)-AFP promoter-T14 × 3-R1] with BglII and XbaI. The AFP enhancer sequence was amplified from HepG2 DNA with AFP enhancer for and AFP enhancer rev by PCR.
**pH188 (pCMV-EYFP-4 × miR-199a-3p target site)** was prepared by inserting 4 × miR-199a-3p target site through annealed miR-199a-3p-1, miR-199a-3p-2, miR-199a-3p-3 and miR-199a-3p-4 into pZ198 (pCMV-EYFP) with HindIII and SalI.
**pH198 (pCMV-EYFP-4 × miR-142 target site)** was prepared by inserting 4 × miR-142 target site through annealed miR-142-1, miR-142-2, miR-142-3 and miR-142-4 into pZ198 (pCMV-EYFP) with HindIII and SalI.
**pH190 (pCMV-EYFP-4 × miR-122 target site)** was prepared by inserting 4 × miR-122 target site through annealed miR-122-1, miR-122-2, miR-122-3 and miR-122-4 into pZ198 (pCMV-EYFP) with HindIII and SalI.
**pH194 (pCMV-EYFP-4 × miR-21 target site)** was prepared by inserting 4 × miR-21 target site through annealed miR-21-1, miR-21-2, miR-21-3 and miR-21-4 into pZ198 (pCMV-EYFP) with HindIII and SalI.
**pH517 (pCMV-EYFP-4 × shRNA FF5 target site)** was prepared by inserting 4 × shRNA FF5 target site through annealed FF5-1, FF5-2, FF5-3 and FF5-4 into pZ198 (pCMV-EYFP) with HindIII and SalI.
**pZ198 (pCMV-EYFP)** was prepared by inserting EYFP into pamCyan-C1 with NheI and HindIII. The EYFP fragment was amplified from pBrainbow1.1M.
**pZ375 (pENTR_L4-5 × UAS-2 × LacO-R1)** was prepared by inserting 5 × UAS-2 × LacO fragment into pZ296 (pENTR_L4-5 × UAS_R1) with XhoI and EcoRI. The fragment was synthesized.
**pB308（pENTR_L4-5 × UAS-2 × tetO-R1）** was prepared by inserting 5 × UAS-2 × tetO fragment into pB307 (pENTR_L4-5 × UAS_R1) with XbaI and EcoRI. The fragment was synthesized.
**pCH009 (pSIREN-U6-shRNA FF4-pCMV-dsRed)** was prepared by inserting oligo annealing shRNA FF4 into pZ010 (pU6-SH17-pCMV-dsRed) by BamHI and EcoRI.
**pCH010 (pSIREN-U6-shRNA FF5-pCMV-dsRed)** was prepared by inserting oligo annealing shRNA FF5 into pZ010 (pU6-SH17-pCMV-dsRed) by BamHI and EcoRI.

**pCH160 (pZD_Seq2-pCAG-Gal4VP16-Seq3)** was prepared by combining pZ250 (pENTR_L4-pCAG-R1) and pCH152 (pENTR_L1-Kozak sequence-Gal4VP16-L2) with pZDonor_1-GTW-2 in a Gateway LR reaction.
**pCH151 (pENTR_L1-Kozak sequence-EYFP-2A-TALER21-4 × FF5 target site-L2)** was prepared by inserting TALER21 into pCH049 (pENTR_L1-Kozak sequence-mKate2-2A-TALER21-4 × FF3 target site-L2) with AgeI and SpeI.
**pT437 (pMM_BsaI-recognizing site 1-Esp3I-CCDB-Esp3I-recognizing site 8-BsaI)** was prepared by inserting BsaI-1-Esp3I-CCDB-Esp3I-8-BsaI fragment into pT433 (pMM_insert MCS) by NotI and NcoI.
**pT450 (pMM_BsaI-recognizing site 10-Esp3I-CCDB-Esp3I-recognizing site 9-BsaI)** was prepared by inserting BsaI-10-Esp3I-CCDB-Esp3I-9-BsaI fragment into pT433 (pMM_insert MCS) by NotI and NcoI.
**pT458 (pMM_BsaI-recognizing site 8-Esp3I-CCDB-Esp3I-recognizing site 10-BsaI)** was prepared by inserting BsaI-8-Esp3I-CCDB-Esp3I-10-BsaI fragment into pT433 (pMM_insert MCS) by NotI and NcoI.
**pH469 (pENTR_L1-tetR-SV40NLS-Krab-4 × miR21 target site-L2)** was prepared by combining tetR-SV40NLS fragment, Krab fragment, 4 × miR-21 target site fragment and pDS016 (pENTR_BsaI-CCDB-BsaI-NLS) by Golden Gate reaction with BsaI. The tetR-SV40NLS fragment was amplified from pH361(pENTR_L1-tetR-EYFP-4 × miR-21 target site-L2) with H160109 and H160106 by PCR. Krab fragment was amplified from pZ348 (pZD_Seq2-pTRE-ZFKrab-2A-mKate2-Seq3) with H160107 and H160108 by PCR. the 4 × miR-21 fragment was digested from pCH153 (pENTR_L1-Kozak sequence-EYFP-2A-TALER9-4 × miR-21 target site-L2) with EcoRI and BamHI.
**pH470 (pZD_Seq1-5 × UAS-2 × LacO-tetR-Krab-4 × miR-21 target site-Seq2)** was prepared by combining pH469 (pENTR_L1-tetR-SV40NLS-krab-4 × miR21 target site-L2) and pZ375 (pENTR_L4-5 × UAS-2 × LacO-R1) with pZDonor_1-GTW-2 in a Gateway LR reaction.
**pH471 (pENTR_L1-EYFP-2A-tetR-Krab-4 × miR-21 target site-L2)** was prepared by combining tetR-SV40NLS fragment, Krab fragment, 4 × miR-21 trarget site fragment and pDS016 (pENTR_BsaI-CCDB-BsaI-NLS) by Golden Gate reaction with BsaI. The tetR-SV40NLS fragment was amplified from pH361 (pENTR_L1-tetR-EYFP-4 × miR-21 target site-L2) with H160105 and H160106 by PCR. Krab fragment was amplified from pZ348 (pZD_Seq2-pTRE-ZFKrab-2A-mKate2-Seq3) with H160107 and H160108 by PCR. the 4 × miR-21 fragment was digested from pCH153 (pENTR_L1-Kozak sequence-EYFP-2A-TALER9-4 × miR-21 target site-L2) with AgeI and BamHI.
**pH472 (pZD_Seq1-5 × UAS-2 × LacO-EYFP-2A-tetR-Krab-4 × miR-21 target site-Seq2)** was prepared by combining pH471 (pENTR_L1-EYFP-2A-tetR-Krab-4 × miR-21 target site-L2) and pZ375 (pENTR_L4-5 × UAS-2 × LacO-R1) with pZDonor_1-GTW-2 in a Gateway LR reaction.
**pH470-FF5-pT458 (pMM_BsaI-recognizing site 8-5 × UAS-2 × LacO-tetR-Krab-4 × shRNA FF5 target site-SV40 poly A- recognizing site 10-BsaI)** was prepared by combining 5 × UAS-2 × LacO-tetR-Krab fragment, 4 × FF5 target site fragment, SV40 poly A fragment and pT458 (pMM_BsaI-recognizing site 8-Esp3I-CCDB-Esp3I-recognizing site 10-BsaI) by Golden Gate with Esp3I. 5 × UAS-2 × LacO-tetR-Krab fragment was amplified from pH470 (pZD_Seq1-5 × UAS-2 × LacO-tetR-Krab-4 × miR-21 target site-Seq2) with H160434 and H160437 by PCR. 4 × FF5 target site fragment was amplified from pCH151 (pENTR_L1-Kozak sequence-EYFP-2A-TALER21-4 × FF5 target site-L2) with H150513 and H150514. SV40 poly A fragment was amplified from pH470 (pZD_Seq1-5 × UAS-2 × LacO-tetR-Krab-4 × miR-21 target site-Seq2) with H160440 and H160441 by PCR.
**pH472-FF5-pT458 (pMM_BsaI-recognizing site 8-5 × UAS-2 × LacO-EYFP-2A-tetR-Krab-4 × shRNA FF5 target site-SV40 poly A- recognizing site 10-BsaI)** was prepared by combining 5 × UAS-2 × LacO-

EYFP-2A-tetR-Krab fragment, 4 × FF5 target site fragment, SV40 poly A fragment and pT458 (pMM_BsaI-recognizeing site 8-Esp3I-CCDB-Esp3I-recognizeing site 10-BsaI) by Golden Gate with Esp3I. 5 × UAS-2 × LacO-EYFP-2A-tetR-Krab fragment was amplified from pH472 (pZD_Seq1-5 × UAS-2 × LacO-tetR-Krab-4 × miR-21 target site-Seq2) with H160434 and H160437. 4 × FF5 target site fragment was amplified from pCH151 (pENTR_L1-Kozak sequence-EYFP-2A-TALER21-4 × FF5 target site-L2) with H150513 and H150514. SV40 poly A fragment was amplified from pH470 (pZD_Seq1-5 × UAS-2 × LacO-tetR-Krab-4 × miR-21 target site-Seq2) with H160440 and H160441 by PCR.
**pH489 (pENTR_L1-E1A-P2A-EBFP-2A-LacI-4 × miR-199a-3p target site-4 × miR-142 target site-L2)** was prepared by combining E1A-P2A fragment, P2A-LacI fragment, 4 × miR-199a-3p target site-4 × miR-142 target site fragment and pDS016 (pENTR_BsaI-CCDB-BsaI-NLS) by Golden Gate with Esp3I. E1A-P2A fragment was amplified from pH363 (pENTR_L1-E1A-2A-EBFP-2A-LacI-4 × miR-199a-3p-4 × miR-142-L2) with H160307 and H160308 by PCR. P2A-EBFP-2A-LacI fragment was amplified from pH363 (pENTR_L1-E1A-2A-EBFP-2A-LacI-4 × miR-199a-3p-4 × miR-142-L2) with H160309 and H160313 by PCR. 4 × miR-199a-3p target site-4 × miR-142 target site fragment was amplified from pH363 (pENTR_L1-E1A-2A-EBFP-2A-LacI-4 × miR-199a-3p-4 × miR-142-L2) with H160314 and H160315 by PCR.
**pH490 (pZD_Seq1-5 × UAS-2 × tetO-E1A-P2A-EBFP-2A-LacI-4 × miR-199a-3p target site -4 × miR-142 target site-Seq2)** was prepared by combining pH489 (pENTR_L1-E1A-P2A-EBFP-2A-LacI-4 × miR-199a-3p target site-4 × miR-142 target site-L2) and pB308（pENTR-L4_5 × UAS-2 × tetO-R1） with pZDonor_1-GTW-2 in a Gateway LR reaction.
**pH490-FF4-pT437 (pMM_BsaI-recognizing site 1-In-Fusion site-5 × UAS-2 × tetO-E1A-P2A-EBFP-2A-LacI-4 × shRNA FF4 target site-recognizing site 8-BsaI)** was prepared by combining 5 × UAS-2 × tetO fragment, E1A-P2A-EBFP-2A-LacI fragment, 4 × shRNA FF4 target site fragment and pT437(pMM_BsaI-recognizing site 1-Esp3I-CCDB-Esp3I-recognizing site 8-BsaI) by Golden Gate with Esp3I. 5 × UAS-2 × tetO fragment was amplified from pH490 (pZD_Seq1-5 × UAS-2 × tetO-E1A-P2A-EBFP-2A-LacI-4 × miR-199a-3p target site -4 × miR-142 target site-Seq2) with H160430 and H160431 by PCR, E1A-P2A-EBFP-2A-LacI fragment was amplified from pH490 (pZD_Seq1-5 × UAS-2 × tetO-E1A-P2A-EBFP-2A-LacI-4 × miR-199a-3p target site -4 × miR-142 target site-Seq2) with H150509 and H150510. 4 × shRNA FF4 target site fragment was amplified from pCH053 (pENTR_L1-Kozak sequence-mKate2-2A-TALER14-4 × shRNA FF4 target site-L2) with H150511 andH150512.The In-Fusion site was prepared for inserting the switch into adenoviral vector in the third step by Gibson.
**pH441 (pENTR_L1-E1A-2A-mGM-CSF-2A-LacI-4 × shRNA FF5 target site-4 × miR-142 target site-L2)** was prepared by inserting mouse GM-CSF fragment into pH351 (pENTR_L1-E1A-2A-EBFP-2A-LacI-4 × shRNA FF5 target site-4 × miR-142 target site) with NcoI and BglII. Mouse GM-CSF sequence was synthesized.
**pH442 (pENTR_L1-E1A-2A-IL-2-2A-LacI-4 × shRNA FF5 target site-4 × miR-142 target site-L2)** was prepared by inserting IL-2 fragment into pH351 (pENTR_L1-E1A-2A-EBFP-2A-LacI-4 × shRNA FF5 target site-4 × miR-142 target site) with NcoI and BglII. IL-2 sequence was synthesized.
**pH490-pT437 (pMM_BsaI-recognizing site 1-In-Fusion site-5 × UAS-2 × tetO-E1A-P2A-EBFP-2A-LacI-4 × miR-199a-3p target site-4 × miR-142 target site-recognizing site 8-BsaI)** was prepared by combining 5 × UAS-2 × tetO fragment, E1A-P2A-EBFP-2A-LacI fragment, 4× miR-199a-3p target site-4 × miR-142 target site and pT437(pMM_BsaI-recognizing site 1-Esp3I-CCDB-Esp3I-recognizing site 8-BsaI) by Golden Gate with Esp3I. 5 × UAS-2 × tetO fragment was amplified from pH490 (pZD_Seq1-5 × UAS-2 × tetO-E1A-P2A-

EBFP-2A-LacI-4 × miR-199a-3p target site -4 × miR-142 target site-Seq2) with H160430 and H160431 by PCR, E1A-P2A-EBFP-2A-LacI fragment was amplified from pH490 (pZD_Seq1-5 × UAS-2 × tetO-E1A-P2A-EBFP-2A-LacI-4 × miR-199a-3p target site -4 × miR-142 target site-Seq2) with H150509 and H150510. 4 × miR-199a-3p target site-4 × miR-142 target site fragment was amplified from pH490 (pZD_Seq1-5 × UAS-2 × tetO-E1A-P2A-EBFP-2A-LacI-4 × miR-199a-3p target site -4 × miR-142 target site-Seq2) with H150511 and H160433 by PCR. The In-Fusion site was prepared for inserting the switch into adenoviral vector in the third step by Gibson.
**pH490-IL-2-pT437 (pMM_BsaI-recognizing site 1-In-Fusion site-5 × UAS-2 × tetO-E1A-P2A-IL-2-2A-LacI-4 × miR-199a-3p target site-4 × miR-142 target site-recognizing site 8-BsaI)** was prepared by combining 5 × UAS-2 × tetO fragment, E1A-half P2A fragment, half P2A-IL-2-2A-LacI fragment, 4 × miR-199a-3p target site-4 × miR-142 target site and pT437 (pMM_BsaI-recognizing site 1-Esp3I-CCDB-Esp3I-recognizing site 8-BsaI) by Golden Gate with Esp3I. 5 × UAS-2 × tetO fragment was amplified from pH490 (pZD_Seq1-5 × UAS-2 × tetO-E1A-P2A-EBFP-2A-LacI-4 × miR-199a-3p target site -4 × miR-142 target site-Seq2) with H160430 and H160431 by PCR, E1A-half P2A fragment was amplified from pH490 (pZD_Seq1-5 × UAS-2 × tetO-E1A-P2A-EBFP-2A-LacI-4 × miR-199a-3p target site -4 × miR-142 target site-Seq2) with H150509 and H160308. Half p2A-IL-2-2A-LacI fragment was amplified from pH442 (pENTR_L1-E1A-2A-IL-2-2A-LacI-4 × shRNA FF5 target site-4 × miR-142 target site-L2) with H160309 and H160313. 4 × miR-199a-3p target site-4 × miR-142 target site fragment was amplified from pH490 (pZD_Seq1-5 × UAS-2 × tetO-E1A-P2A-EBFP-2A-LacI-4 × miR-199a-3p target site -4 × miR-142 target site-Seq2) with H160314 and H160433 by PCR. The In-Fusion site was prepared for inserting the switch into adenoviral vector in the third step by Gibson.
**pH490-mGM-CSF-pT437 (pMM_BsaI-recognizing site 1-In-Fusion site-5 × UAS-2 × tetO-E1A-P2A-mGM-CSF-2A-LacI-4 × miR-199a-3p target site-4 × miR-142 target site-recognizing site 8-BsaI)** was prepared by combining 5 × UAS-2 × tetO fragment, E1A-half P2A fragment, half P2A-mGM-CSF-2A-LacI fragment, 4 × miR-199a-3p target site-4 × miR-142 target site and pT437 (pMM_BsaI-recognizing site 1-Esp3I-CCDB-Esp3I-recognizing site 8-BsaI) by Golden Gate with Esp3I. 5 × UAS-2 × tetO fragment was amplified from pH490 (pZD_Seq1-5 × UAS-2 × tetO-E1A-P2A-EBFP-2A-LacI-4 × miR-199a-3p target site -4 × miR-142 target site-Seq2) with H160430 and H160431 by PCR. E1A-half P2A fragment was amplified from pH490 (pZD_Seq1-5 × UAS-2 × tetO-E1A-P2A-EBFP-2A-LacI-4 × miR-199a-3p target site -4 × miR-142 target site-Seq2) with H150509 and H160308. Half p2A-mGM-CSF-2A-LacI fragment was amplified from pH441 (pENTR_L1-E1A-2A-IL-2-2A-LacI-4 × shRNA FF5 target site-4 × miR-142 target site-L2) with H160309 and H160313. 4 × miR-199a-3p target site-4 × miR-142 target site fragment was amplified from pH490 (pZD_Seq1-5 × UAS-2 × tetO-E1A-P2A-EBFP-2A-LacI-4 × miR-199a-3p target site -4 × miR-142 target site-Seq2) with H160314 and H160433 by PCR. The In-Fusion site was prepared for inserting the switch into adenoviral vector in the third step by Gibson.
**pH490-anti-PD-1 scFv-PT437 (pMM_BsaI-recognizing site 1-In-Fusion site-5 × UAS-2 × tetO-E1A-P2A-anti-PD-1 scFv-2A-LacI-4 × miR-199a-3p target site-4 × miR-142 target site-recognizing site 8-BsaI)** was prepared by combining 5 × UAS-2 × tetO fragment, E1A-half P2A fragment, half P2A-anti-PD-1 scfv-2A-LacI fragment, 4 × miR-199a-3p target site-4 × miR-142 target site and pT437 (pMM_BsaI-recognizing site 1-Esp3I-CCDB-Esp3I-recognizing site 8-BsaI) by Golden Gate with Esp3I. 5 × UAS-2 × tetO fragment was amplified from pH490 (pZD_Seq1-5 × UAS-2 × tetO-E1A-P2A-EBFP-2A-LacI-4 × miR-199a-3p target site -4 × miR-142 target site-Seq2) with H160430 and H160431 by PCR, E1A-half P2A fragment was amplified from

pH490 (pZD_Seq1-5 × UAS-2 × tetO-E1A-P2A-EBFP-2A-LacI-4 × miR-199a-3p target site -4 × miR-142 target site-Seq2) with H150509 and H160308. Half p2A-anti-PD-1 scFv-2A-LacI fragment was made by overlap PCR with H160309, H160421, H160420 and H160313. Anti-PD-1 scFv was synthesized. 4 × miR-199a-3p target site-4 × miR-142 target site fragment was amplified from pH490 (pZD_Seq1-5 × UAS-2x × tetO-E1A-P2A-EBFP-2A-LacI-4 × miR-199a-3p target site -4 × miR-142 target site-Seq2) with H160314 and H160433 by PCR. The In-Fusion site was prepared for inserting the switch into adenoviral vector in the third step by Gibson.
**pH490-anti-PD-L1 scFv-pT437 (pMM_BsaI-recognizing site 1- In-Fusion site-5 × UAS-2 × tetO-E1A-P2A-anti-PD-L1 scFv-2A-LacI-4 × miR-199a-3p target site-4 × miR-142 target site-recognizing site 8-BsaI)** was prepared by combining 5 × UAS-2 × tetO fragment, E1A-half P2A fragment, half P2A-anti-PD-L1 scfv-2A-LacI fragment, 4 × miR-199a-3p target site-4 × miR-142 target site and pT437 (pMM_BsaI-recognizing site 1-Esp3I-CCDB-Esp3I-recognizing site 8-BsaI) by Golden Gate with Esp3I. 5 × UAS-2 × tetO fragment was amplified from pH490 (pZD_Seq1-5 × UAS-2 × tetO-E1A-P2A-EBFP-2A-LacI-4 × miR-199a-3p target site -4 × miR-142 target site-Seq2) with H160430 and H160431 by PCR, E1A-half P2A fragment was amplified from pH490 (pZD_Seq1-5 × UAS-2 × tetO-E1A-P2A-EBFP-2A-LacI-4 × miR-199a-3p target site -4 × miR-142 target site-Seq2) with H150509 and H160308. Half p2A-anti-PD-L1 scFv-2A-LacI fragment was made by overlap PCR with H160309, H160421, H160420 and H160313. Anti-PD-L1 scFv was synthesized. 4 × miR-199a-3p target site-4 × miR-142 target site fragment was amplified from pH490 (pZD_Seq1-5 × UAS-2 × tetO-E1A-P2A-EBFP-2A-LacI-4 × miR-199a-3p target site -4 × miR-142 target site-Seq2) with H160314 and H160433 by PCR. The In-Fusion site was prepared for inserting the switch into adenoviral vector in the third step by Gibson.
**pH470-pT458 (pMM_BsaI-recognizing site 8-5 × UAS-2 × LacO-tetR-Krab-4 × miR-21 target site-SV40 poly A- recognizing site 10-BsaI)** was prepared by combining 5 × UAS-2 × LacO-tetR-Krab-4 × miR-21 target site-SV40 poly A fragment and pT458 (pMM_BsaI-recognizing site 8-Esp3I-CCDB-Esp3I-recognizing site 10-BsaI) with Esp3I. The 5 × UAS-2 × LacO-tetR-Krab-4 × miR-21 target site-SV40 poly A fragment was amplified from pH470 (pZD_Seq1-5 × UAS-2 × LacO-tetR-Krab-4 × miR-21 target site-Seq2) with H160434 and H160437 by PCR.
**pH358-pT450 (pMM_BsaI-recognizing site 10-AFP enhancer domain A-AFP enhancer domain B-point mutation -199(G to A) promoter-Gal4VP16-rb global terminator-In-Fusion site-recognizing site 1-BsaI)** was prepared by combining liver specific promoter fragment, Gal4VP16 fragment, rb global terminator fragment and pT450 (pMM_BsaI-recognizing site 10-Esp3I-CCDB-Esp3I-recognizing site 9-BsaI) with Esp3I. The AFP promoter was amplified from pH358 [pZD_Seq1-AFP enhancer domain A-AFP enhancer domain B-point mutation -199(G to A) promoter-Gal4VP16_Seq2] with H160448 and H160449, Gal4VP16 fragment was amplified from pH358 [pZD_Seq1-AFP enhancer domain A-AFP enhancer domain B-point mutation -199 (G to A) promoter-Gal4VP16_Seq2] with H160450 and H160451, the rb global terminator fragment was amplified from pH358 [pZD_Seq1-AFP enhancer domain A-AFP enhancer domain B-point mutation -199(G to A) promoter-Gal4VP16_Seq2] with H160452 and H160453 by PCR. The In-Fusion site was prepared for inserting the switch into adenoviral vector in the third step by Gibson.
**pH518 (pZD_L1-recognizing site 1-BsaI-CCDB-BsaI-recognizing site 9_L2')** was prepared by inserting the L2' fragment into pT1-9 (pZD_L1-recognizing site 1 -BsaI-CCDB-BsaI-recognizing site 9_L2) with SalI and XhoI. The L2' fragment was amplified from Adenoviral vector (Invitrogen) with H160715 and H160716 by PCR.

**pH519 [pZD_L1-In-Fusion site-5 × UAS-2 × tetO-E1A-P2A-EBFP-2A-LacI-4 × miR-199a-3p target site-4 × miR-142 target site-5 × UAS-2 × LacO-tetR-Krab-4 × miR-21 target site-SV40 poly A-AFP enhancer domain A-AFP enhancer domain B-point mutation -199 (G to A) promoter-Gal4VP16-rb global terminator-In-Fusion site-L2']** was prepared by combining pH490-pT437 (pMM_BsaI-recognize site 1-In-Fusion site-5 × UAS-2 × tetO-E1A-P2A-EBFP-2A-LacI-4 × miR-199a-3p target site-4 × miR-142 target site-recgnize site 8-BsaI), pH470-pT458 (pMM_BsaI-recognizing site 8-5 × UAS-2xLacO-tetR-Krab-4 × miR-21 target site-SV40 poly A-recognizing site 10-BsaI), pH358-pT450 (pMM_BsaI-recognizing site 10-AFP enhancer domain A-AFP enhancer domain B-point mutation -199 (G to A) promoter-Gal4VP16-rb global terminator-In-Fusion site-recognizing site 1-BsaI) and pH518 (pZD_L1-recognizing site 1-BsaI-CCDB-BsaI-recognizing site 9_L2') by Golden Gate with BsaI.
**pH520 [pZD_L1-In-Fusion site-5 × UAS-2 × tetO-E1A-P2A-IL-2-2A-LacI-4 × miR-199a-3p target site-4 × miR-142 target site-5 × UAS-2 × LacO-tetR-Krab-4 × miR-21 target site-SV40 poly A-AFP enhancer domain A-AFP enhancer domain B-point mutation -199 (G to A) promoter-Gal4VP16-rb global terminator-In-Fusion site-L2']** was prepared by combining pH490-IL-2-pT437 (pMM_BsaI-recognizing site 1-In-Fusion site-5 × UAS-2 × tetO-E1A-P2A-IL-2-2A-LacI-4 × miR-199a-3p target site-4 × miR-142 target site-recognizing site 8-BsaI), pH470-pT458 (pMM_BsaI-recognizing site 8-5 × UAS-2 × LacO-tetR-Krab-4 × miR-21 target site-SV40 poly A-recognizing site 10-BsaI), pH358-pT450 (pMM_BsaI-recognizing site 10-AFP enhancer domain A-AFP enhancer domain B-point mutation -199 (G to A) promoter-Gal4VP16-rb global terminator-In-Fusion site-recognizing site 1-BsaI) and pH518 (pZD_L1-recognizing site 1-BsaI-CCDB-BsaI-recognizing site 9_L2') by Golden Gate with BsaI.
**pH521 [pZD_L1-In-Fusion site-5 × UAS-2 × tetO-E1A-P2A-mGM-CSF-2A-LacI-4 × miR-199a-3p target site-4 × miR-142 target site-5 × UAS-2 × LacO-tetR-Krab-4 × miR-21 target site-SV40 poly A-AFP enhancer domain A-AFP enhancer domain B-point mutation -199 (G to A) promoter-Gal4VP16-rb global terminator-In-Fusion site-L2']** was prepared by combining pH490-mGM-CSF-pT437 (pMM_BsaI-recognizing site 1-In-Fusion site-5 × UAS-2 × tetO-E1A-P2A-mGM-CSF-2A-LacI-4 × miR-199a-3p target site-4 × miR-142 target site-recognizing site 8-BsaI), pH470-pT458 (pMM_BsaI-recognizing site 8-5 × UAS-2 × LacO-tetR-Krab-4 × miR-21 target site-SV40 poly A-recognizing site 10-BsaI), pH358-pT450[pMM_BsaI-recognizing site 10-AFP enhancer domain A-AFP enhancer domain B-point mutation -199 (G to A) promoter-Gal4VP16-rb global terminator-In-Fusion site-recognizing site 1-BsaI] and pH518 (pZD_L1-recognizing site 1-BsaI-CCDB-BsaI-recognizing site 9-L2') by Golden Gate with BsaI.
**pH522 [pZD_L1-In-Fusion site-5 × UAS-2 × tetO-E1A-P2A-anti-PD-1 scFv-2A-LacI-4 × miR-199a-3p target site-4 × miR-142 target site-5 × UAS-2 × LacO-tetR-Krab-4 × miR-21 target site-SV40 poly A-AFP enhancer domain A-AFP enhancer domain B-point mutation -199 (G to A) promoter-Gal4VP16-rb global terminator-In-Fusion site-L2']** was prepared by combining pH490-anti-PD-1 scFv-pT437 (pMM_BsaI-recognizing site 1-In-Fusion site-5 × UAS-2 × tetO-E1A-P2A-anti-PD-1 csFv-2A-LacI-4 × miR-199a-3p target site-4 × miR-142 target site-recognizing site 8-BsaI), pH470-pT458 (pMM_BsaI-recognizing site 8-5 × UAS-2 × LacO-tetR-Krab-4 × miR-21 target site-SV40 poly A- recognizing site 10-BsaI),pH358-pT450 [pMM_BsaI-recognizing site 10-AFP enhancer domain A-AFP enhancer domain B-point mutation -199(G to A) promoter-Gal4VP16-rb global terminator-In-Fusion site-recognizing site 1-BsaI] and pH518 (pZD_L1-recognizing site 1-BsaI-CCDB-BsaI-recognizing site 9-L2') by Golden Gate with BsaI.
**pH523 [pZD_L1-In-Fusion site-5 × UAS-2 × tetO-E1A-P2A-anti-PD-L1 scFv-2A-LacI-4 × miR-199a-3p target site-4 × miR-142 target site-5 × UAS-2 × LacO-tetR-Krab-4 × miR-21 target site-SV40 poly A-AFP**

**enhancer domain A-AFP enhancer domain B-point mutation -199 (G to A) promoter-Gal4VP16-rb global terminator-In-Fusion site-L2']** was prepared by combining pH490-anti-PD-L1 scFv-pT437 (pMM_BsaI-recognizing site 1-In-Fusion site-5 × UAS-2 × tetO-E1A-P2A-anti-PD-L1 csFv-2A-LacI-4 × miR-199a-3p target site-4 × miR-142 target site-recognizing site 8-BsaI), pH470-pT458 (pMM_BsaI-recognizing site 8-5 × UAS-2 × LacO-tetR-Krab-4 × miR-21 target site-SV40 poly A- recognizing site 10-BsaI),pH358-pT450 [pMM_BsaI-recognizing site 10-AFP enhancer domain A-AFP enhancer domain B-point mutation -199 (G to A) promoter-Gal4VP16-rb global terminator-In-Fusion site-recognizing site 1-BsaI] and pH518 (pZD_L1-recognizing site 1-BsaI-CCDB-BsaI-recognizing site 9-L2') by Golden Gate with BsaI.
**pH524 (ITR-package signal-In-Fusion site-5xUAS-2xtetO-E1A-P2A-EBFP-2A-LacI-4x miR-199a-3p target site-miR-142 target site-5xUAS-2xLacO-tetR-Krab-4xmiR-21 target site-SV40 poly A-AFP enhancer domain A-AFP enhancer domain B-point mutation -199(G to A) promoter-Gal4VP16-rb global terminator-In-Fusion site-ITR)** was prepared by cmbining pH519 (pZD_L1-In-Fusion site-5xUAS-2xtetO-E1A-P2A-EBFP-2A-LacI-4x miR-199a-3p target site-miR-142 target site-5xUAS-2xLacO-tetR-Krab-4xmiR-21 target site-SV40 poly A-AFP enhancer domain A-AFP enhancer domain B-point mutation -199(G to A) promoter-Gal4VP16-rb global terminator-In-Fusion site_L2') and adenoviral vector (Invitrogen) in a Gateway LR reaction.
**pH525 [ITR-package signal-In-Fusion site-5 × UAS-2 × tetO-E1A-P2A-IL-2-2A-LacI-4 × miR-199a-3p target site-4 × miR-142 target site-5 × UAS-2 × LacO-tetR-Krab-4 × miR-21 target site-SV40 poly A-AFP enhancer domain A-AFP enhancer domain B-point mutation -199 (G to A) promoter-Gal4VP16-rb global terminator-In-Fusion site-ITR]** was prepared by combining pH520 [pZD_L1-In-Fusion site-5 × UAS-2 × tetO-E1A-P2A-IL-2-2A-LacI-4 × miR-199a-3p target site-4 × miR-142 target site-5 × UAS-2 × LacO-tetR-Krab-4 × miR-21 target site-SV40 poly A-AFP enhancer domain A-AFP enhancer domain B-point mutation -199 (G to A) promoter-Gal4VP16-rb global terminator-In-Fusion site-L2'] and adenoviral vector (Invitrogen) in a Gateway LR reaction.
**pH526 [ITR-package signal-In-Fusion site-5 × UAS-2 × tetO-E1A-P2A-mGM-SCF-2A-LacI-4 × miR-199a-3p target site-4 × miR-142 target site-5 × UAS-2 × LacO-tetR-Krab-4 × miR-21 target site-SV40 poly A-AFP enhancer domain A-AFP enhancer domain B-point mutation -199 (G to A) promoter-Gal4VP16-rb global terminator-In-Fusion site-ITR]** was prepared by combining pH521 [pZD_L1-In-Fusion site-5 × UAS-2 × tetO-E1A-P2A-mGM-CSF-2A-LacI-4 × miR-199a-3p target site-4 × miR-142 target site-5 × UAS-2 × LacO-tetR-Krab-4 × miR-21 target site-SV40 poly A-AFP enhancer domain A-AFP enhancer domain B-point mutation -199 (G to A) promoter-Gal4VP16-rb global terminator-In-Fusion site-L2'] and adenoviral vector (Invitrogen) in a Gateway LR reaction.
**pH527 [ITR-package signal-In-Fusion site-5 × UAS-2 × tetO-E1A-P2A-anti-PD-1 scfv-2A-LacI-4 × miR-199a-3p target site-4 × miR-142 target site-5 × UAS-2 × LacO-tetR-Krab-4 × miR-21 target site-SV40 poly A-AFP enhancer domain A-AFP enhancer domain B-point mutation -199 (G to A) promoter-Gal4VP16-rb global terminator-In-Fusion site-ITR]** was prepared by cmbining pH522 [pZD_L1-In-Fusion site-5 × UAS-2 × tetO-E1A-P2A-anti-PD-1 scFv-2A-LacI-4 × miR-199a-3p target site-4 × miR-142 target site-5 × UAS-2 × LacO-tetR-Krab-4 × miR-21 target site-SV40 poly A-AFP enhancer domain A-AFP enhancer domain B-point mutation -199 (G to A) promoter-Gal4VP16-rb global terminator-In-Fusion site-L2'] and adenoviral vector (Invitrogen) in a Gateway LR reaction.
**pH528 [ITR-package signal-In-Fusion site-5 × UAS-2 × tetO-E1A-P2A-anti-PD-L1 scfv-2A-LacI-4 × miR-199a-3p target site-4 × miR-142 target site-5 × UAS-2 × LacO-tetR-Krab-4 × miR-21 target site-**

**SV40 poly A-AFP enhancer domain A-AFP enhancer domain B-point mutation -199 (G to A) promoter-Gal4VP16-rb global terminator-In-Fusion site-ITR]** was prepared by combining pH523 (pZD_L1-In-Fusion site-5 × UAS-2 × tetO-E1A-P2A-anti-PD-L1 scFv-2A-LacI-4 × miR-199a-3p target site-4 × miR-142 target site-5 × UAS-2 × LacO-tetR-Krab-4 × miR-21 target site-SV40 poly A-AFP enhancer domain A-AFP enhancer domain B-point mutation -199 (G to A) promoter-Gal4VP16-rb global terminator-In-Fusion site-L2') and adenoviral vector (Invitrogen) in a Gateway LR reaction.
**pH199 [In-Fusion site-pCMV-EYFP-SV40 polyA-In-Fusion site]** was prepared by combining In-Fusion site-pCMV-EYFP-SV40 polyA-In-Fusion site fragment and pZ198 (pCMV-EYFP-SV40 polyA) with PciI and MluI. The In-Fusion site-pCMV-EYFP-SV40 polyA-In-Fusion site fragment was amplified from pZ198 with In-Fusion for and In-Fusion rev by PCR.
**pH529 [ITR-package signal-In-Fusion site-pCMV-EYFP-SV40 polyA-In-Fusion site-ITR]** was prepared by combining In-Fusion site-pCMV-EYFP-SV40 polyA-In-Fusion site fragment and adenoviral vector (Clontech) in a Gibson reaction. In-Fusion site-pCMV-EYFP-SV40 polyA-In-Fusion site fragment was digested from pH199 (In-Fusion site-pCMV-EYFP-SV40 polyA-In-Fusion site) with PciI and MluI.

Supplementary Table 3 Transfection Configuration.

Plasmid DNA used in Fig. 2a	Amount						
pDT7004	100ng	100ng					
phEF1a-EBFP	100ng	100ng					
AFP enhancer domain A-domain B-AFP point mutation promoter-Gal4VP16	100ng						
pCAG-Gal4VP16		100ng					
5×UAS-miniCMV-EYFP-2A-TALER9-4 × miR-21 target site	100ng	100ng					
Plasmid DNA used in Fig. 2b	Amount						
pDT7004	100ng	100ng	100ng	100ng	100ng		
phEF1a-EBFP	100ng	100ng	100ng	100ng	100ng		
pCMV-EYFP-4 × shRNA FF5 target site	100ng						
pCMV-EYFP-4 × miR-199a-3p target site		100ng					
pCMV-EYFP-4 × miR-122 target site			100ng				
pCMV-EYFP-4 × miR-21 target site				100ng			

pCMV-EYFP-4 × miR-142 target site					100ng		
Plasmid DNA used in Fig. 2c	Amount						
pDT7004	100ng	100ng	100ng	100ng	100ng	100ng	
phEF1a-mKate2	100ng	100ng	100ng	100ng	100ng	100ng	
5 × UAS-tetO × 2-E1A-P2A-EBFP-2A-LacI-4 × shRNA FF4 target site	100ng	100ng	100ng	100ng	100ng	100ng	
5 × UAS-LacO × 2-tetR-Krab-4 × shRNA FF5 target site	100ng	100ng	100ng				
5 × UAS-LacO × 2-EYFP-2A-tetR-Krab-4 × shRNA FF5 target site				100ng	100ng	100ng	
pCAG-Gal4VP16	100ng	100ng	100ng	100ng	100ng	100ng	
pSIREN_U6-shRNA FF4-pCMV-dsRed		100ng			100ng		
pSIREN_U6-shRNA FF5-pCMV-dsRed			100ng			100ng	
Plasmid DNA used in Fig. 3a	Amount						
pDT7004	450ng	350ng	350ng				
pCMV-EYFP		100ng					
phEF1a-EBFP			100ng				
AFP enhancer domain A-AFP enhancer domain B-AFP-promoter-Gal4VP16							
5 × UAS-tetO × 2-kozak-E1A-P2A-EBFP-2A-LacI-4 × shRNA FF4 target site							
5 × UAS-tetO × 2-E1A-P2A-EBFP-2A-LacI-4 × miR-142 target site-4 × miR-199a-3p target site							
Seq1- 5 × UAS-LacO × 2-tetR-Krab-4 × miR-21 target site							
pCAG-Gal4VP16							
Plasmid DNA used in Fig. 3a	Amount						
pDT7004	100ng	130ng	140ng	145ng	149ng	150ng	150ng
pCMV-EYFP	100ng	100ng	100ng	100ng	100ng	100ng	100ng
phEF1a-EBFP							

AFP enhancer domain A-AFP enhancer domain B-AFP-promoter-Gal4VP16	100ng	100ng	100ng	100ng	100ng	100ng	100ng
5 × UAS-tetO × 2-kozak-E1A-P2A-EBFP-2A-LacI-4 × shRNA FF4 target site	100ng	100ng	100ng	100ng	100ng	100ng	100ng
5 × UAS-tetO × 2-E1A-P2A-EBFP-2A-LacI-4 × miR-142 target site-4 × miR-199a-3p target site							
Seq1- 5 × UAS-LacO x 2-tetR-Krab-4 × miR-21 target site							
pCAG-Gal4VP16	50ng	20ng	10ng	5ng	1ng	0.5ng	
Plasmid DNA used in Fig. 3a	Amount						
pDT7004	100ng	130ng	140ng	145ng	149ng	150ng	150ng
pCMV-EYFP	100ng	100ng	100ng	100ng	100ng	100ng	100ng
phEF1a-EBFP							
AFP enhancer domain A-AFP enhancer domain B-AFP promoter-Gal4VP16	100ng	100ng	100ng	100ng	100ng	100ng	100ng
5 × UAS-tetO × 2-kozak-E1A-P2A-EBFP-2A-LacI-4 × shRNA FF4 target site							
5 × UAS-tetO × 2-E1A-P2A-EBFP-2A-LacI-4 × miR-142 target site-4 × miR-199a-3p target site	100ng	100ng	100ng	100ng	100ng	100ng	100ng
Seq1- 5 × UAS-LacO × 2-tetR -Krab-4 × miR-21 target site							
pCAG-Gal4VP16	50ng	20ng	10ng	5ng	1ng	0.5ng	
Plasmid DNA used in Fig. 3a	Amount						
pDT7004		30ng	40ng	45ng	49ng	50ng	
pCMV-EYFP	100ng	100ng	100ng	100ng	100ng	100ng	100ng
phEF1a-EBFP							
AFP enhancer domain A-AFP enhancer domain B-Gal4VP16	100ng	100ng	100ng	100ng	100ng	100ng	100ng
5 × UAS-tetO × 2-kozak-E1A-P2A-EBFP-2A-LacI-shRNA FF4 target site							
5 × UAS-tetO × 2-E1A-P2A-EBFP-2A-LacI-4 × miR-142 target site-4 × miR-199a-3p target site	100ng	100ng	100ng	100ng	100ng	100ng	100ng
Seq1- 5 × UAS-LacO × 2-tetR-sv40NLS-Krab-4 × miR-21 target site	100ng	100ng	100ng	100ng	100ng	100ng	100ng
pCAG-Gal4VP16	50ng	20ng	10ng	5ng	1ng	0.5ng	
Plasmid DNA used in Supplementary Fig. 1b	Amount						
pDT7004	100ng	100ng	100ng	100ng	100ng		

phEF1a-EBFP	100ng	100ng	100ng	100ng	100ng		
AFP enhancer-AFP point mutation promoter-Gal4VP16	100ng						
AFP 821bp enhancer-AFP point mutation promoter-Gal4VP16		100ng					
AFP enhancer domain A-domain B-AFP point mutation promoter-Gal4VP16			100ng				
AFP enhancer domain A-AFP point mutation promoter-Gal4VP16				100ng			
AFP enhancer domain B-AFP point mutation promoter-Gal4VP16					100ng		
5 × UAS-miniCMV-EYFP-2A-TALER9-4 × miR-21 target site	100ng	100ng	100ng	100ng	100ng		
Plasmid DNA used in Supplementary Fig. 1c	Amount						
pDT7004	200ng	100ng	100ng				
pCMV-EYFP		100ng		100ng			
phEF1a-EBFP			100ng	100ng	100ng	100ng	100ng
pCMV-EYFP-4×miR-199a-3p target site					100ng		
pCMV-EYFP-4×miR-21 target site						100ng	
AFP enhancer A Enhancer domain B-AFP promoter-EYFP							100ng

Supplementary Table 4 Parameters used in the model of oncolytic virus therapeutic system.

Parameter	Description	Value	Remark
γ	The proliferation rate of uninfected cancer cells	0.3	Fitted from Supplementary Fig. 9c (HepG2: 0.38, Huh7: 0.30)
K	The saturation amount of cancer cells	10	Assumed
κ	The infection rate of free viruses	0.002	Fitted from Supplementary Fig. 9c (HepG2: 0.0025, Huh7: 0.0015)
δ	The lysis rate of infected cancer cells	0.2	Fitted from Supplementary Fig. 9c (HepG2: 0.14, Huh7: 0.33)
α	The average number of new viruses released from the lysis of infected cancer cells	1800	Fitted from Supplementary Fig. 9c (HepG2: 1670, Huh7: 1860)

ω	The removing rate of free viruses (degradation, infection into normal cells, etc)	3	Assumed, and discussed in Supplementary Fig. 9d
p_T	The killing rate of cancer cells presenting the cancer-specific antigen	0.05	Assumed
p_V	The killing rate of cancer cells presenting the virus-specific antigen	0.05	Assumed
c_T	The proliferation rate of cytotoxic lymphocytes activated by the cancer-specific antigen	0.5	Assumed, and discussed in Supplementary Fig. 10b
c_V	The proliferation rate of cytotoxic lymphocytes activated by the virus-specific antigen	0.5	Assumed, and discussed in Supplementary Fig. 10b
β	The degradation rate of cytotoxic lymphocytes	0.1	Assumed
ϕ	The ratio of cytotoxic lymphocytes that are not suppressed in the microenvironment	0	Assumed
K_F	The amount of immune effectors to reach half activation	1	Assumed
λ_I	The production rate of immune effectors loaded in viral genome	0	Assumed, and discussed in Supplementary Fig. 11c
λ_F	The degradation rate of immune effectors	0.5	Assumed
