# Supplementary material for: Oncolytic adenovirus programmed by synthetic gene circuit for cancer immunotherapy
Source: Nat Commun. 2019 Oct 22;10:4801. doi: 10.1038/s41467-019-12794-2 (PMC6805884; doi:10.1038/s41467-019-12794-2)
Supplement: Supplementary file 2 — Reporting Summary [file 41467_2019_12794_MOESM2_ESM.pdf]

nature research

Corresponding author(s): Zhen Xie

Reporting Summary

Nature Research wishes to improve the reproducibility of the work that we publish. This form provides structure for consistency and transparency in reporting. For further information on Nature Research policies, see Authors & Referees and the Editorial Policy Checklist.

Statistical parameters

When statistical analyses are reported, confirm that the following items are present in the relevant location (e.g. figure legend, table legend, main text, or Methods section).

n/a	Confirmed	
☐	☒	The exact sample size (*n*) for each experimental group/condition, given as a discrete number and unit of measurement
☐	☒	An indication of whether measurements were taken from distinct samples or whether the same sample was measured repeatedly
☐	☒	The statistical test(s) used AND whether they are one- or two-sided<br>*Only common tests should be described solely by name; describe more complex techniques in the Methods section.*
☐	☒	A description of all covariates tested
☐	☒	A description of any assumptions or corrections, such as tests of normality and adjustment for multiple comparisons
☐	☒	A full description of the statistics including central tendency (e.g. means) or other basic estimates (e.g. regression coefficient) AND variation (e.g. standard deviation) or associated estimates of uncertainty (e.g. confidence intervals)
☐	☒	For null hypothesis testing, the test statistic (e.g. *F*, *t*, *r*) with confidence intervals, effect sizes, degrees of freedom and *P* value noted<br>*Give P values as exact values whenever suitable.*
☒	☐	For Bayesian analysis, information on the choice of priors and Markov chain Monte Carlo settings
☒	☐	For hierarchical and complex designs, identification of the appropriate level for tests and full reporting of outcomes
☒	☐	Estimates of effect sizes (e.g. Cohen's *d*, Pearson's *r*), indicating how they were calculated
☐	☒	Clearly defined error bars<br>*State explicitly what error bars represent (e.g. SD, SE, CI)*

Our web collection on statistics for biologists may be useful.

Software and code

Policy information about availability of computer code

Data collection	A custom MATLAB script was used
Data analysis	A custom MATLAB script was used

For manuscripts utilizing custom algorithms or software that are central to the research but not yet described in published literature, software must be made available to editors/reviewers upon request. We strongly encourage code deposition in a community repository (e.g. GitHub). See the Nature Research guidelines for submitting code & software for further information.

Data

Policy information about availability of data

All manuscripts must include a data availability statement. This statement should provide the following information, where applicable:

- Accession codes, unique identifiers, or web links for publicly available datasets
- A list of figures that have associated raw data
- A description of any restrictions on data availability

Plasmids information was listed in Supplementary Information. The deep sequencing data at endogenous target sites will be deposited to NCBI database. Accession code will be available before publication.

Field-specific reporting

Please select the best fit for your research. If you are not sure, read the appropriate sections before making your selection.

☒ Life sciences ☐ Behavioural & social sciences ☐ Ecological, evolutionary & environmental sciences

For a reference copy of the document with all sections, see nature.com/authors/policies/ReportingSummary-flat.pdf

Life sciences study design

All studies must disclose on these points even when the disclosure is negative.

Sample size	Experiments were performed in biological triplicate n=3 unless otherwise noted.
Data exclusions	No data was excluded.
Replication	All attempts at replication were successful, and standard deviations were within expected ranges.
Randomization	Different cell passages were used for each biological replicate.
Blinding	Not applicable, as samples were processed identically through standard protocol that should not bias outcomes.

Reporting for specific materials, systems and methods

Materials & experimental systems

n/a	Involved in the study
☒	☐ Unique biological materials
☐	☒ Antibodies
☐	☒ Eukaryotic cell lines
☒	☐ Palaeontology
☐	☒ Animals and other organisms
☒	☐ Human research participants

Methods

n/a	Involved in the study
☒	☐ ChIP-seq
☐	☒ Flow cytometry
☒	☐ MRI-based neuroimaging

Antibodies

Antibodies used	anti-mouse CD3 antibody (BioLegend) ; anti-mouse IFN-γ antibody (Clone XMG1.2, BD Bioscience);anti-mouse Ki-67 antibody (Clone 16A8, Biolegend).;Anti-CD45.2 antibody (Clone 104, Biolegend)
Validation	all the antibody related data had been provide on the manuscript

Eukaryotic cell lines

Policy information about cell lines

Cell line source(s)	HEK293FT was purchased from Life Technology
Authentication	Cells were authenticated by the supplier.
Mycoplasma contamination	HEK293FT cells tested negative for mycoplasma.
Commonly misidentified lines (See ICLAC register)	No commonly misidentified cell lines were used.

Animals and other organisms

Policy information about studies involving animals; ARRIVE guidelines recommended for reporting animal research

Laboratory animals	six week old female BALB/c nude mice and six week old female C57BL/6 mice were used in this study

Wild animals	*Provide details on animals observed in or captured in the field; report species, sex and age where possible. Describe how animals were caught and transported and what happened to captive animals after the study (if killed, explain why and describe method; if released, say where and when) OR state that the study did not involve wild animals.*
Field-collected samples	*For laboratory work with field-collected samples, describe all relevant parameters such as housing, maintenance, temperature, photoperiod and end-of-experiment protocol OR state that the study did not involve samples collected from the field.*

Flow Cytometry

Plots

Confirm that:

- [x] The axis labels state the marker and fluorochrome used (e.g. CD4-FITC).
- [x] The axis scales are clearly visible. Include numbers along axes only for bottom left plot of group (a 'group' is an analysis of identical markers).
- [x] All plots are contour plots with outliers or pseudocolor plots.
- [x] A numerical value for number of cells or percentage (with statistics) is provided.

Methodology

Sample preparation	Cells were trypsinized 3 days after transfection and centrifuged at 300 g for 10 min at 4 °C. The supernatant was removed, and the cells were resuspended in 1× PBS that did not contain calcium or magnesium.
Instrument	Fortessa analyzer (BD Biosciences)
Software	Flowjo 7.6.1 was used for fluorescence-activated cell sorting (FACS) analysis.
Cell population abundance	HEK293 luciferase + in transfected cells were typically over 50% of the population.
Gating strategy	Negative control (unstained) and fluorophore-positive cells were used to establish gates for each cell type.

- [ ] Tick this box to confirm that a figure exemplifying the gating strategy is provided in the Supplementary Information.
